# Pregnancy after bariatric surgery and adverse perinatal outcomes: A systematic review and meta-analysis

**DOI:** 10.1371/journal.pmed.1002866

**Published:** 2019-08-06

**Authors:** Zainab Akhter, Judith Rankin, Dries Ceulemans, Lem Ngongalah, Roger Ackroyd, Roland Devlieger, Rute Vieira, Nicola Heslehurst

**Affiliations:** 1 Institute of Health & Society, Newcastle University, Newcastle upon Tyne, United Kingdom; 2 Department of Obstetrics and Gynaecology, University Hospitals Leuven, Leuven, Belgium; 3 Department of Surgery, Sheffield Teaching Hospitals, Sheffield, United Kingdom; 4 Institute of Health Sciences Research, University of Aberdeen, Aberdeen, United Kingdom; Cornell University, UNITED STATES

## Abstract

**Background:**

Women who undergo bariatric surgery prior to pregnancy are less likely to experience comorbidities associated with obesity such as gestational diabetes and hypertension. However, bariatric surgery, particularly malabsorptive procedures, can make patients susceptible to deficiencies in nutrients that are essential for healthy fetal development. The objective of this systematic review and meta-analysis is to investigate the association between pregnancy after bariatric surgery and adverse perinatal outcomes.

**Methods and findings:**

Searches were conducted in Medline, Embase, PsycINFO, CINAHL, Scopus, and Google Scholar from inception to June 2019, supplemented by hand-searching reference lists, citations, and journals. Observational studies comparing perinatal outcomes post-bariatric surgery to pregnancies without prior bariatric surgery were included. Outcomes of interest were perinatal mortality, congenital anomalies, preterm birth, postterm birth, small and large for gestational age (SGA/LGA), and neonatal intensive care unit (NICU) admission. Pooled effect sizes were calculated using random-effects meta-analysis. Where data were available, results were subgrouped by type of bariatric surgery. We included 33 studies with 14,880 pregnancies post-bariatric surgery and 3,979,978 controls. Odds ratios (ORs) were increased after bariatric surgery (all types combined) for perinatal mortality (1.38, 95% confidence interval [CI] 1.03–1.85, *p* = 0.031), congenital anomalies (1.29, 95% CI 1.04–1.59, *p* = 0.019), preterm birth (1.57, 95% CI 1.38–1.79, *p* < 0.001), and NICU admission (1.41, 95% CI 1.25–1.59, *p* < 0.001). Postterm birth decreased after bariatric surgery (OR 0.46, 95% CI 0.35–0.60, *p* < 0.001). ORs for SGA increased (2.72, 95% CI 2.32–3.20, *p* < 0.001) and LGA decreased (0.24, 95% CI 0.14–0.41, *p* < 0.001) after gastric bypass but not after gastric banding. Babies born after bariatric surgery (all types combined) weighed over 200 g less than those born to mothers without prior bariatric surgery (weighted mean difference −242.42 g, 95% CI −307.43 to −177.40 g, *p* < 0.001). There was low heterogeneity for all outcomes (*I*^*2*^ < 40%) except LGA. Limitations of our study are that as a meta-analysis of existing studies, the results are limited by the quality of the included studies and available data, unmeasured confounders, and the small number of studies for some outcomes.

**Conclusions:**

In our systematic review of observational studies, we found that bariatric surgery, especially gastric bypass, prior to pregnancy was associated with increased risk of some adverse perinatal outcomes. This suggests that women who have undergone bariatric surgery may benefit from specific preconception and pregnancy nutritional support and increased monitoring of fetal growth and development. Future studies should explore whether restrictive surgery results in better perinatal outcomes, compared to malabsorptive surgery, without compromising maternal outcomes. If so, these may be the preferred surgery for women of reproductive age.

**Trial registration:**

PROSPERO CRD42017051537.

## Abstract 2

Zainab Akhter and colleagues reveal that bariatric surgery for women prior to pregnancy can result in adverse outcomes for newborns, including intensive care admission and preterm birth

## Author summary

**Why was this study done?:**

- Obesity during pregnancy increases the risk of health complications for both mother and baby.
- Bariatric surgery before pregnancy improves obesity-related problems for the mother but reduces the absorption of micronutrients that are needed for healthy fetal development.
- This research aimed to investigate whether bariatric surgery is associated with adverse outcomes for the baby.

**What did the researchers do and find?:**

- This systematic review included 33 studies that investigated perinatal outcomes among women with previous bariatric surgery compared to women without previous bariatric surgery.
- Meta-analysis identified a significant increase in odds of perinatal mortality, congenital anomalies, preterm birth, and neonatal intensive care unit admission but a decrease in odds of postterm birth after bariatric surgery.
- The odds of small babies were increased and the odds of large babies were decreased after malabsorptive bariatric surgery types, but there was no change for restrictive bariatric surgery types.

**What do these findings mean?:**

- Bariatric surgery, in particular malabsorptive types of surgery, seems associated with an increased risk of some adverse perinatal outcomes, which suggests a link with nutrition.
- Women of reproductive age undergoing bariatric surgery are a high-risk group and require specialised preconception and antenatal nutritional support to achieve the best outcomes for both mothers and babies.

## Introduction

Obesity is a global public health challenge with over 650 million adults affected worldwide, and prevalence continues to rise, making obesity the most common medical condition in women of reproductive age [1,2]. Maternal obesity, defined as prepregnancy body mass index (BMI) ≥ 30 kg/m^2^, has severe implications for both mother and baby. Maternal risks include higher likelihood of gestational diabetes, preeclampsia, and cesarean section [3]. For the neonate, there is increased risk of pre- and postterm birth, small and large for gestational (SGA/LGA), congenital anomalies, and perinatal mortality [3,4]. Interventions to reduce maternal obesity are important not only to improve pregnancy outcomes but also to reduce the long-term health burden on the mother and offspring, including cardiovascular disease and insulin resistance [5].

Bariatric surgery is the most effective treatment for long-term weight loss, and over half of surgeries are performed on women of reproductive age [6,7]. Women who undergo bariatric surgery prior to pregnancy are less likely to experience comorbidities associated with obesity, such as gestational diabetes and hypertension [8]. However, micronutrient deficiencies are increased after bariatric surgery and may therefore have implications for fetal environment [9]. Maternal deficiencies in folate, iron, and vitamin D, for example, are all linked with adverse perinatal outcomes including neural tube defects, preterm birth, and low birth weight [10]. Malabsorptive procedures such as Roux-en-Y gastric bypass (RYGB) and biliopancreatic diversion (BPD) reduce the absorption of micronutrients because part of the small intestine is bypassed, whereas restrictive procedures such as laparoscopic adjustable gastric banding (LAGB) and sleeve gastrectomy (SG) reduce stomach capacity [11]. There have been multiple case reports of congenital anomalies occurring after malabsorptive procedures because of maternal malnutrition; however, the evidence from observational studies is conflicting [12].

Previous meta-analyses on pregnancy after bariatric surgery have focused on maternal outcomes, and there is limited evidence on perinatal outcomes other than size for gestational age and preterm birth [8,13,14]. The aim of this systematic review and meta-analysis was to compare adverse perinatal outcomes among women who underwent bariatric surgery prior to pregnancy with those who had not. When possible, the difference in effect size between malabsorptive and restrictive procedures was explored.

## Methods

### Search strategy and selection criteria

Searches were conducted in Medline, Embase, PsycINFO, CINAHL, Scopus, Google Scholar, and relevant e-journals from inception to June 3, 2019. We included observational studies published in the English language, involving women who had undergone bariatric surgery prior to pregnancy, and compared them to women without a history of bariatric surgery. We included studies that combined all types of bariatric surgery or provided data for RYGB, LAGB, SG, or BPD separately. The following perinatal outcomes were included: perinatal mortality (including stillbirth), congenital anomalies, preterm birth, postterm birth, SGA, LGA, neonatal intensive care unit (NICU) admission, birth weight, and gestational age.

The search strategy (S1 Table) included a mixture of keywords and MeSH headings: (pregnan* or mother* or matern*) and (bariatric surgery or weight loss and surgery or gastric bypass or gastric band* or sleeve or biliopancreatic diversion or LAGB or RYGB) and (death or mortality or newborn* or fetal or congenital or stillbirth or defect* or perinatal or obstetric or neonat* or outcome* or birth). Reference lists and citations were searched for all included primary studies and for relevant reviews identified by the database searches. Authors were contacted if additional data were required for inclusion in meta-analysis. Screening, data extraction, and quality assessment were carried out in duplicate.

This review was conducted in line with the PRISMA and MOOSE guidelines (S1 PRISMA Checklist) [15,16]. The protocol is published on PROSPERO (CRD42017051537).

### Data analysis

The Cochrane Cohort Study data extraction tool was adapted to meet the requirements of this review. Study characteristics extracted included study design, study location, type of bariatric surgery, and control group. Frequencies, effect sizes, and confidence intervals (CIs) of adverse perinatal outcomes were also extracted. For continuous outcomes, means and standard deviations were extracted. When multiple studies reported data from the same cohort with the same participant inclusion criteria, the decision was made to include the study with the larger sample size for the exposed group. Studies with duplicate data were only included if they reported different perinatal outcomes and were therefore included in separate meta-analysis. The Newcastle-Ottawa quality assessment scale was used to appraise the quality of the included studies out of a maximum of eight points (S1 Fig). The studies were assessed for representativeness of the exposed cohort, selection of the nonexposed cohort, ascertainment of exposure and outcome, study design and analysis, and adequacy of follow-up.

A meta-analysis was used to calculate a pooled odds ratio (OR) and 95% CI when there were at least three studies reporting the same outcome. For continuous perinatal outcomes, a weighted mean difference (WMD) and 95% CI were calculated. DerSimonian and Laird random-effects model was used to take clinical heterogeneity into account such as unreported differences between surgical procedures (e.g., technique and limb length) and different levels of patient postsurgery and preconception care. When a study reported data on multiple control groups, a hierarchy was developed to firstly include the most comparable BMI group to the postbariatric patient, which was prepregnancy BMI matched, then obesity. When there was evidence of moderate heterogeneity (*I*^*2*^ > 40%), subgroup analysis by type of surgery or comparison group, as defined a priori, was carried out if three or more studies existed for each group. Any remaining heterogeneity was explored through meta-regression for factors including location, sample size, publication date, and quality. Publication bias was investigated using Egger’s test and funnel plots. For studies reporting adjusted results, their crude and adjusted ORs were compared to determine whether adjustments affected the effect size. Sensitivity analysis was performed for each meta-analysis by excluding one study at a time to identify the effect of any individual study on the pooled effect size and between-study heterogeneity. All analyses were conducted in Stata/SE 15.0.

## Results

### Study characteristics

**Fig 1:**
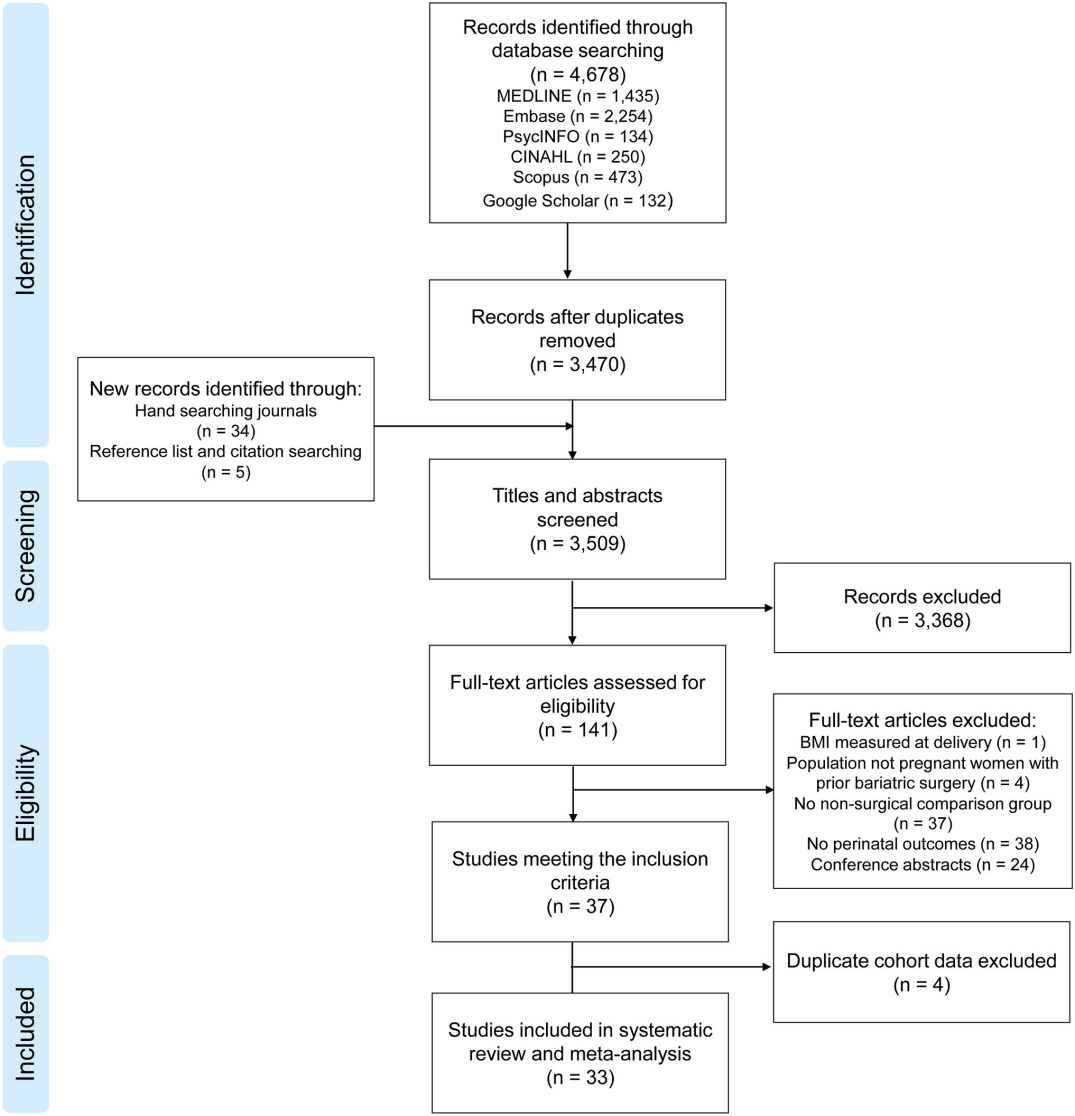
PRISMA flowchart of included studies. BMI, body mass index.

Database searches identified 3,470 results for title and abstract screening, of which 141 studies underwent full-text assessment (Fig 1). The kappa statistic for inter-rater agreement of study inclusion between authors was 0.84 (scores > 0.81 are considered excellent) [17]. Thirty-seven studies met the inclusion criteria, but four were excluded because they reported the same cohort, participant inclusion criteria, and outcomes as another study [18–21]. This resulted in 33 studies that reported original data on perinatal outcomes (14,880 pregnancies after bariatric surgery and 3,979,978 pregnancies without bariatric surgery, Table 1). Fifteen of the included studies were conducted in Europe, 10 were conducted in the United States, three in Israel, two in each Australia and Brazil, and one in Canada. Studies were published between 1998 and 2018. All studies scored over five out of eight for quality, with 20 studies scoring at least seven (S2 Table). Many studies conducted more than one analysis with multiple surgery types or control groups. Sixteen analyses combined all bariatric surgery patients, whereas 14 studies were restricted to RYGB, six analyses included only LAGB, one included only SG, and one included BPD. Nine analyses compared women’s postsurgical pregnancies to pre/early-pregnancy BMI–matched controls, and 14 used obesity controls (which were ≥30 kg/m^2^, 35 kg/m^2^, or 40 kg/m^2^) in line with their relevant bariatric surgery guidelines, or matched for presurgical BMI. Eleven analyses compared pregnancies before and after bariatric surgery, nine compared outcomes to the general population, and five used healthy BMI as the control group.

**Table 1:** Table of studies included in the systematic review and meta-analysis.

Author, publication year, country	Study period	Exposed groups*	Comparison groups*	Perinatal outcomes reported
Adams et al. 2015 [22], USA	Bariatric surgery between 1979 and 2011	(1) 764 pregnancies after RYGB (2) 2,666 pregnancies after RYGB	(1) 764 pregnancies matched for ppBMI (2) 10,447 pregnancies before RYGB	Birth weight Congenital anomalies Gestational age LGA Postterm birth Preterm birth Stillbirth SGA
Belogolovkin et al. 2012 [23], USA	Delivery between 2004 and 2007	293 pregnancies after bariatric surgery	656,353 general population pregnancies	Birth weight Gestational age Macrosomia Preterm birth SGA
Berglind et al. 2014 [24], Sweden	Bariatric surgery between 1980 and 2006	124 pregnancies after bariatric surgery	124 pregnancies before bariatric surgery	Birth weight Gestational age
Berlac et al. 2014 [25], Denmark	Bariatric surgery between January 1996 and June 2011	415 pregnancies after RYGB	827 pregnancies matched for ppBMI 829 healthy BMI 20–24 kg/m^2^ pregnancies	Congenital anomalies NICU admission Stillbirth
Burke et al. 2010 [26], USA	Bariatric surgery between 2002 and 2006	354 pregnancies after bariatric surgery	346 pregnancies matched for presurgery BMI	LGA Stillbirth
Chevrot et al. 2016 [27], France	Delivery between January 1, 2004, and December 31, 2013	(1) 139 pregnancies after bariatric surgery (2) 58 pregnancies after RYGB (2) 81 pregnancies after LAGB or SG	(1) 139 pregnancies matched for presurgery BMI (2) 139 pregnancies matched for ppBMI	Birth weight LGA NICU admission Preterm birth SGA
Dell’Agnolo et al. 2011[28], Brazil	Pregnancy between 1999 and 2008	41 pregnancies after bariatric surgery	14 pregnancies before bariatric surgery	Low birth weight Preterm birth
Dixon et al. 2005 [29], Australia	Bariatric surgery between January 1, 1995, and August 31, 2003	79 pregnancies after LAGB	79 pregnancies with obesity > 35 kg/m^2^ 40 pregnancies before LAGB 61,000 general population pregnancies	Birth weight Low birth weight Macrosomia Preterm birth
Ducarme et al. 2007 [30], France	Delivery between January 2004 and October 2006	13 pregnancies after LAGB	414 pregnancies with obesity > 30kg/m^2^	Gestational age Low birth weight Macrosomia Preterm birth
Feichtinger et al. 2016 [31], Austria	Pregnancy between January 2007 and January 2016	76 pregnancies after RYGB	76 pregnancies with obesity > 30 kg/m^2^ 76 pregnancies matched for ppBMI 76 healthy BMI 18–25 kg/m^2^ pregnancies	LGA NICU admission SGA
Gascoin et al. 2017 [9], France	Delivery between March 1, 2008, and October 31, 2012	56 pregnancies after RYGB	56 nonobesity pregnancies	Birth weight
Goldman et al. 2016 [32], USA	Bariatric surgery between 2002 and 2012	(1) 12 pregnancies after RYGB (2) 14 pregnancies after LAGB	(1)(2) 14 pregnancies with obesity (eligible for bariatric surgery) (1) 36 pregnancies before RYGB (2) 28 pregnancies before LAGB	Birth weight Preterm birth
Hammeken et al. 2017 [33], Denmark	Delivery between January 1, 2010, and December 31, 2013	151 pregnancies after RYGB	151 pregnancies matched for ppBMI	Birth weight Gestational age LGA NICU admission SGA
Johansson et al. 2015 [34], Sweden	Bariatric surgery between 2006 and 2011	596 pregnancies after bariatric surgery	2,356 pregnancies matched for presurgery BMI	Congenital anomalies LGA Preterm birth SGA Stillbirth
Josefsson et al. 2013 [35], Sweden	Mothers born between 1973 and 1983	318 pregnancies after bariatric surgery	244,294 general population pregnancies	Congenital anomalies
Josefsson et al. 2011 [36], Sweden	Mothers born between 1973 and 1983	126 pregnancies after bariatric surgery	188,500 general population pregnancies	Birth weight Gestational age LGA Preterm birth SGA
Kjaer et al. 2013 [37], Denmark	Delivery between January 2004 and December 2010	(1) 339 pregnancies after bariatric surgery (2) 286 pregnancies after RYGB	(1)(2) 1,277 pregnancies matched for ppBMI	LGA Postterm birth Preterm birth SGA
Lapolla et al. 2010 [38], Italy	Bariatric surgery between September 1993 and December 2005	(1) 83 pregnancies after LAGB (2) 27 pregnancies after LAGB	(1) 120 pregnancies with obesity > 40 kg/m^2^ (1) 858 healthy BMI (criteria NR) pregnancies (2) 27 pregnancies before LAGB	Birth weight Gestational age LGA NICU admission Preterm birth SGA
Lesko and Peaceman 2012[39], USA	Delivery between December 1, 2005, and December 1, 2009	70 pregnancies after bariatric surgery	140 pregnancies matched for presurgery BMI 140 pregnancies matched for ppBMI	Macrosomia NICU admission Preterm birth Stillbirth SGA
Machado et al. 2017 [40], Brazil	Pregnancy between March 2008 and March 2012	30 pregnancies after RYGB	60 pregnancies matched for ppBMI	Birth weight Gestational age SGA
Marceau et al. 2004 [41], Canada	Bariatric surgery before 2000	251 pregnancies after BPD	1,577 pregnancies before BPD	Birth weight Congenital anomalies Gestational age LGA SGA Stillbirth
Parent et al. 2017 [42], USA	Delivery between January 1, 1980, and May 30, 2013	1,859 pregnancies after bariatric surgery	8,437 general population pregnancies	Congenital anomalies LGA NICU admission Preterm birth SGA Stillbirth
Parker et al. 2016 [43], USA	Delivery in 2012	1,585 pregnancies after bariatric surgery	185,120 pregnancies with obesity > 30 kg/m^2^	LGA SGA Stillbirth
Patel et al. 2008 [44], USA	Delivery between 2003 and 2006	26 pregnancies after RYGB	66 pregnancies with obesity > 30 kg/m^2^ 188 nonobesity BMI < 30 kg/m^2^pregnancies	Birth weight Congenital anomalies Gestational age Macrosomia Postterm birth Preterm birth SGA
Roos et al. 2013 [45], Sweden	Delivery between 1992 and 2009	2,534 pregnancies after bariatric surgery	12,468 pregnancies matched for ppBMI 1,740,140 general population pregnancies	LGA Preterm birth SGA Stillbirth
Rottenstreich et al. 2018 [46], Israel	Delivery between 2006 and 2016	119 pregnancies after SG	119 pregnancies matched for presurgery BMI	Congenital anomalies LGA NICU admission Preterm birth SGA
Shai et al. 2014 [47], Israel	Delivery between 1988 and 2010	326 pregnancies after bariatric surgery	1,612 pregnancies with obesity > 30 kg/m^2^	Preterm birth
Skull et al. 2004 [48], Australia	Bariatric surgery between 1996 and 2003	49 pregnancies after LAGB	31 pregnancies before LAGB	Birth weight
Stentebjerg et al. 2017 [49], Denmark	Delivery between November 2007 and October 2013	71 pregnancies after RYGB	57,970 general population pregnancies	Birth weight Gestational age Preterm birth
Stephansson et al. 2018 [50], Sweden	Delivery between 1 January 2006 and 31 December 2013	1,431 pregnancies after bariatric surgery	4,476 pregnancies matched for presurgery BMI 798,338 general population pregnancies	Postterm birth
Wax et al. 2008 [51], USA	NR	38 pregnancies after RYGB	76 general population pregnancies	Birth weight Congenital anomalies Gestational age Macrosomia NICU admission Postterm birth Preterm birth SGA
Weintraub et al. 2008 [52], Israel	Delivery between 1988 and 2006	507 pregnancies after bariatric surgery	301 pregnancies before bariatric surgery	Birth weight Congenital anomalies Gestational age IUGR Macrosomia Stillbirth
Wittgrove et al. 1998 [53], USA	NR	36 pregnancies after RYGB	23 pregnancies before RYGB	Macrosomia Preterm birth

The term ‘bariatric surgery’ is used when a study combined all types of surgery or did not specify a surgery type.

*Some studies reported multiple exposed groups and multiple comparison groups. In the case of multiple exposed groups, numbers indicate which comparison group was used. There are no numbers when a single exposed group was compared to all listed comparison groups.

Abbreviations: BMI, body mass index; BPD, biliopancreatic diversion; IUGR, intrauterine growth restriction; LAGB, laparoscopic adjustable gastric banding; LGA, large for gestational age; NICU, neonatal intensive care unit; NR, not reported; ppBMI, prepregnancy BMI; RYGB, Roux-en-Y gastric bypass; SG, sleeve gastrectomy; SGA, small for gestational age.

### Perinatal mortality and congenital anomalies

**Fig 2:**
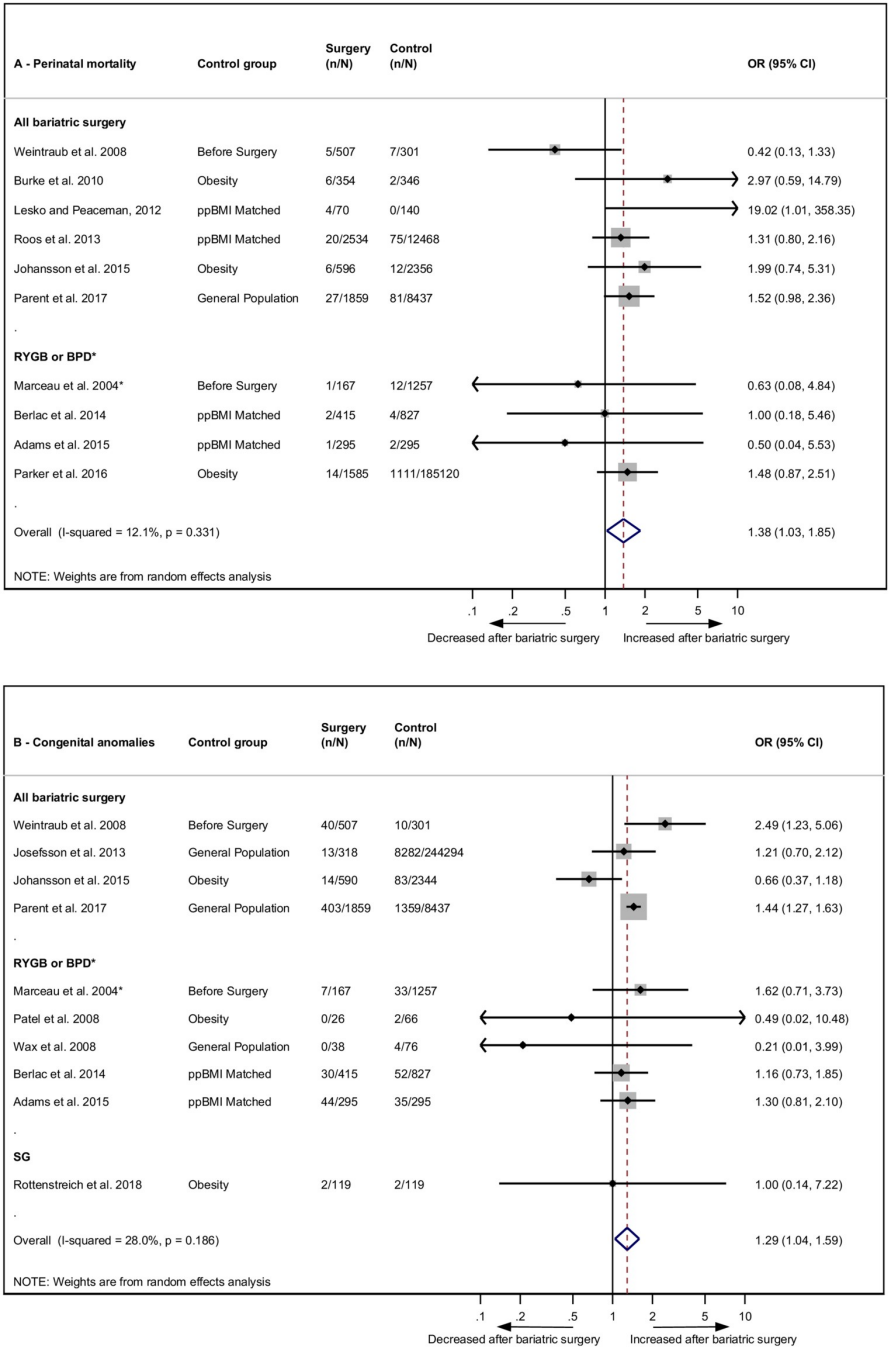
Perinatal mortality and congenital anomalies after bariatric surgery meta-analysis. Association between maternal bariatric surgery and (A) perinatal mortality (includes stillbirth) and (B) congenital anomalies. Studies are presented as Author, year. The forest plots are stratified by type of surgery. *n* = cases of perinatal mortality or congenital anomalies. *N* = total group size. *BPD only. BPD, biliopancreatic diversion; CI, confidence interval; OR, odds ratio; ppBMI, prepregnancy body mass index matched; RYGB, Roux-en-Y gastric bypass; SG, sleeve gastrectomy.

Perinatal mortality or stillbirth was reported in 10 studies. The pooled odds were significantly increased post-bariatric surgery compared to women without prior bariatric surgery (OR 1.38, 95% CI 1.03–1.85, *p* = 0.031) (Fig 2A) [22,25,26,34,39,41–43,45,52]. Ten studies reported on congenital anomalies, which were also found to have significantly increased odds post-bariatric surgery (OR 1.29, 95% CI 1.04–1.59, *p* = 0.019) (Fig 2B) [22,25,34,35,41,42,44,46,51,52]. There was no significant heterogeneity for either outcome (*I*^2^ = 12.1%, 95% CI 0.0–53.1, *p* = 0.331 and *I*^2^ = 28%, 95% CI 0.0–65.5, *p* = 0.186, respectively).

### Gestational age

**Fig 3:**
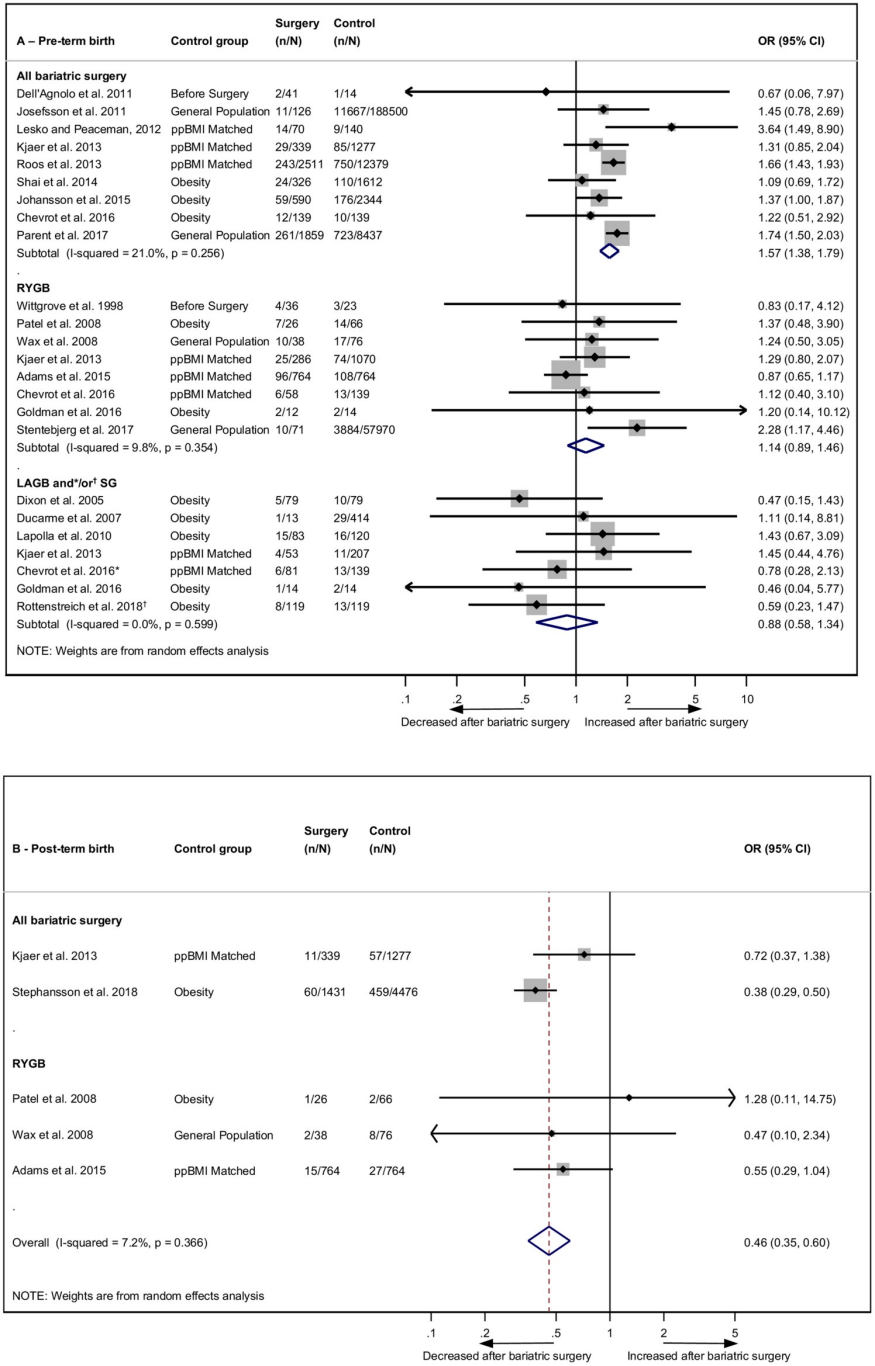
Preterm and postterm birth after bariatric surgery meta-analysis. Association between maternal bariatric surgery and (A) preterm birth (<37 weeks) and (B) postterm birth (>41 or >42 weeks). Studies are presented as Author, year. The forest plots are stratified by type of surgery, with separate pooled OR (95% CI) when subgroup analysis was possible. *n* = cases of preterm or postterm birth. *N* = total group size. *LAGB and SG. ^†^SG only. CI, confidence interval; LAGB, laparoscopic adjustable gastric banding; OR, odds ratio; ppBMI, prepregnancy body mass index matched; RYGB, Roux-en-Y gastric bypass; SG, sleeve gastrectomy.

Preterm birth was reported in 20 studies, with 19 eligible for meta-analysis [22,27–30,32,34,36–39,42,44–47,49,51,53]. The overall odds of preterm birth were significantly increased post-bariatric surgery compared to women without prior bariatric surgery (OR 1.35, 95% CI 1.14–1.60, *p* = 0.001) (S2 Fig). There was significant heterogeneity (*I*^2^ = 50.1%, 95% CI 15.3–70.6, *p* = 0.007), which remained significant after subgroup analyses by control group but was reduced after subgrouping by type of surgery (Fig 3A). There were significantly increased odds of preterm birth after bariatric surgery in the ‘all bariatric surgery’ group (OR 1.57, 95% CI 1.38–1.79, *p* < 0.001). The association was not significant for subgroups ‘RYGB’ (OR 1.14, 95% CI 0.89–1.46, *p* = 0.289) or ‘LAGB or SG’ (OR 0.88, 95% CI 0.58–1.34, *p* = 0.565). The study excluded from the meta-analysis because of lack of crude data reported an adjusted OR for preterm birth of 1.43 (95% CI 1.01–2.03) post-bariatric surgery (*n* = 293) compared to general population controls (*n* = 656,353) [23]. Postterm birth was reported in five studies, and the odds more than halved after bariatric surgery (OR 0.46, 95% CI 0.35–0.60, *p* < 0.001) (Fig 3B) [22,37,44,50,51]. There was no significant heterogeneity (*I*^*2*^ = 7.2%, 95% CI 0.0–80.7, *p* = 0.366).

Despite the results of increased preterm birth and decreased postterm birth, the WMD of 13 studies reporting continuous gestational age did not reach statistical significance (WMD −0.16 weeks, 95% CI −0.38 to 0.06, *p* = 0.156) (S3 Fig) [22–24,30,33,36,38,40,41,44,49,51,52]. Heterogeneity between studies was substantial and did not reduce with subgroup analyses for type of bariatric surgery. Meta-regression revealed that the following factors did not contribute to heterogeneity: type of surgery, control group, publication year, continent, sample size, or quality score (S3A Table).

### Size for gestational age and birth weight

**Fig 4:**
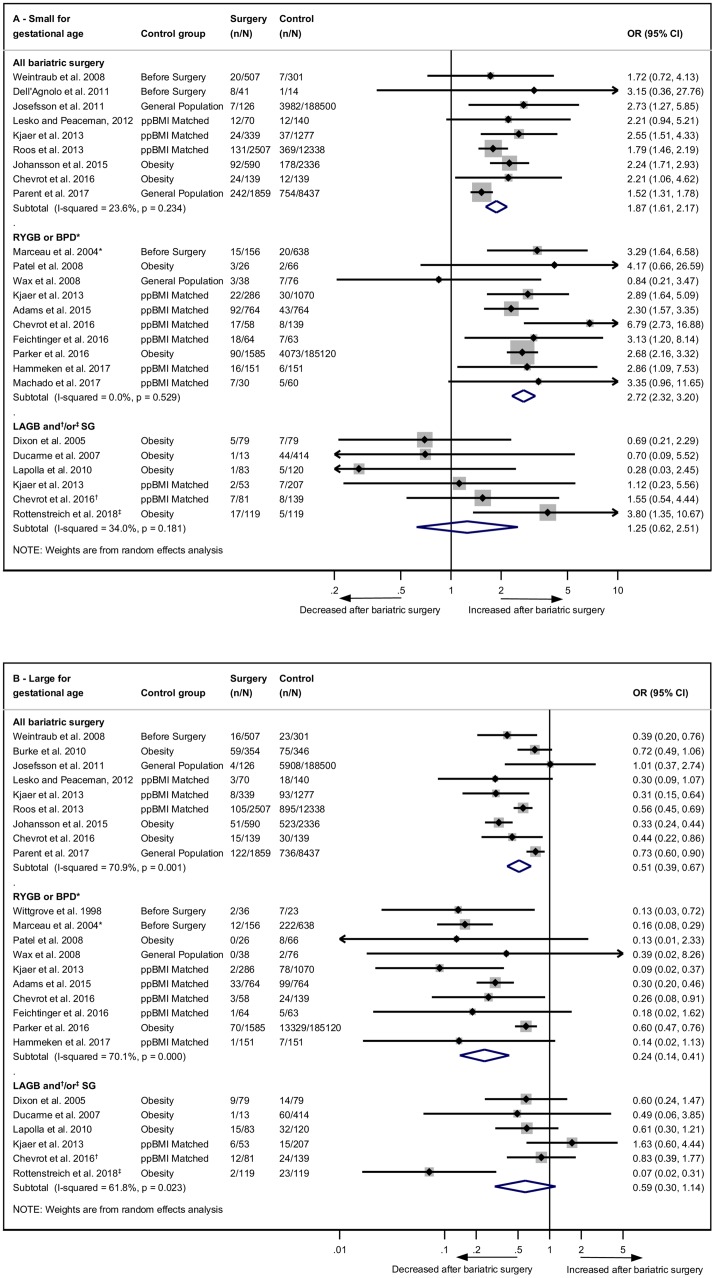
Size for gestational age after bariatric surgery meta-analysis. Association between maternal bariatric surgery and (A) small for gestational age (includes low birth weight < 2,500 g for three studies) and (B) large for gestational age (includes macrosomia > 4,000 g for seven studies). Studies are presented as Author, year. Results are subgrouped by type of surgery. *n* = cases of small or large for gestational age. *N* = total group size. *BPD only. ^†^LAGB and SG. ^‡^SG only. BPD, biliopancreatic diversion; CI, confidence interval; LAGB, laparoscopic adjustable gastric banding; OR, odds ratio; ppBMI, prepregnancy body mass index matched; RYGB, Roux-en-Y gastric bypass; SG, sleeve gastrectomy.

SGA, intrauterine growth restriction, and low birth weight were investigated in 22 studies, and 21 of these were eligible for meta-analysis [22,27–31,33,34,36–46,51,52]. The odds of an SGA baby post-bariatric surgery were more than doubled (OR 2.13, 95% CI 1.80–2.52, *p* < 0.001) (S4 Fig). There was significant evidence of heterogeneity (*I*^2^ = 47.0%, 95% CI 11.8–68.2, *p* = 0.009), which was reduced by subgroup analyses by surgery type (Fig 4A). Odds of SGA were significantly increased for the ‘all bariatric surgery’ group (OR 1.87, 95% CI 1.61–2.17, *p* < 0.001) and were further increased for ‘RYGB or BPD’ (OR 2.72, 95% CI 2.32–3.20, *p* < 0.001). There was no association between SGA and ‘LAGB or SG’ (OR 1.25, 95% CI 0.62–2.51, *p* = 0.533). The study excluded from the meta-analysis reported an adjusted OR of 2.69 (95% CI 1.96–3.69) post-bariatric surgery (*n* = 293) compared to general population controls (*n* = 656,353) [23].

LGA and macrosomia were investigated in 22 studies, and 21 were eligible for meta-analysis [22,26,27,29–31,33,34,36–39,41–46,51–53]. The ORs of an LGA baby post-bariatric surgery were more than halved (0.42, 95% CI 0.34–0.54, *p* < 0.001) (S5 Fig). There was substantial evidence of heterogeneity (*I*^*2*^ = 69.5%, 95% CI 52.4–80.5, *p* < 0.001). Subgroup analyses by type of surgery identified that the ‘RYGB or BPD’ group was associated with the biggest decrease in odds of LGA (OR 0.24, 95% CI 0.14–0.41, *p* < 0.001), in comparison with ‘all bariatric surgery’ (OR 0.51, 95% CI 0.39–0.67, *p* < 0.001), and ‘LAGB or SG’, which was not significant (OR 0.59, 95% CI 0.30–1.14, *p* = 0.116) (Fig 4B). Heterogeneity did not decrease in these subgroup analyses. Meta-regression revealed that sample size was significantly contributing to heterogeneity (residual *I*^*2*^ = 61.21, coefficient = 0.249, *p* = 0.031) (S3B Table). The study excluded from the meta-analysis reported an adjusted OR of 0.03 (95% CI 0.01–0.21) for LGA post-bariatric surgery (*n* = 293) compared to general population controls (*n* = 656,353) [23].

Birth weight mean and standard deviation for babies born after maternal bariatric surgery and controls were reported in 17 studies [9,22–24,27,29,32,33,36,38,40,41,44,48,49,51,52]. WMD was significantly lower post-bariatric surgery (WMD −242.42 g, 95% CI −307.43 g to −177.40 g, *p* < 0.001) (S6 Fig). Heterogeneity was substantial (*I*^*2*^ = 75.7%, 95% CI 61.1–84.8, *p* < 0.001) but reduced after subgroup analyses by surgery type. RYGB resulted in the largest reduction in birth weight (WMD −226.10 g, 95% CI −273.43 g to −178.78 g, *p* < 0.001), compared with ‘all bariatric surgery’ (WMD −223.71 g, 95% CI −273.68 g to −173.74 g, *p* < 0.001), and ‘LAGB’, for which the reduction was not significant (WMD −135.14 g, 95% CI −289.17 g to 18.90 g, *p* = 0.086). One study investigated only BPD, for which the mean difference was −500 g (95% CI −570.85 g to −429.15 g, *p* < 0.001).

### NICU admission

**Fig 5:**
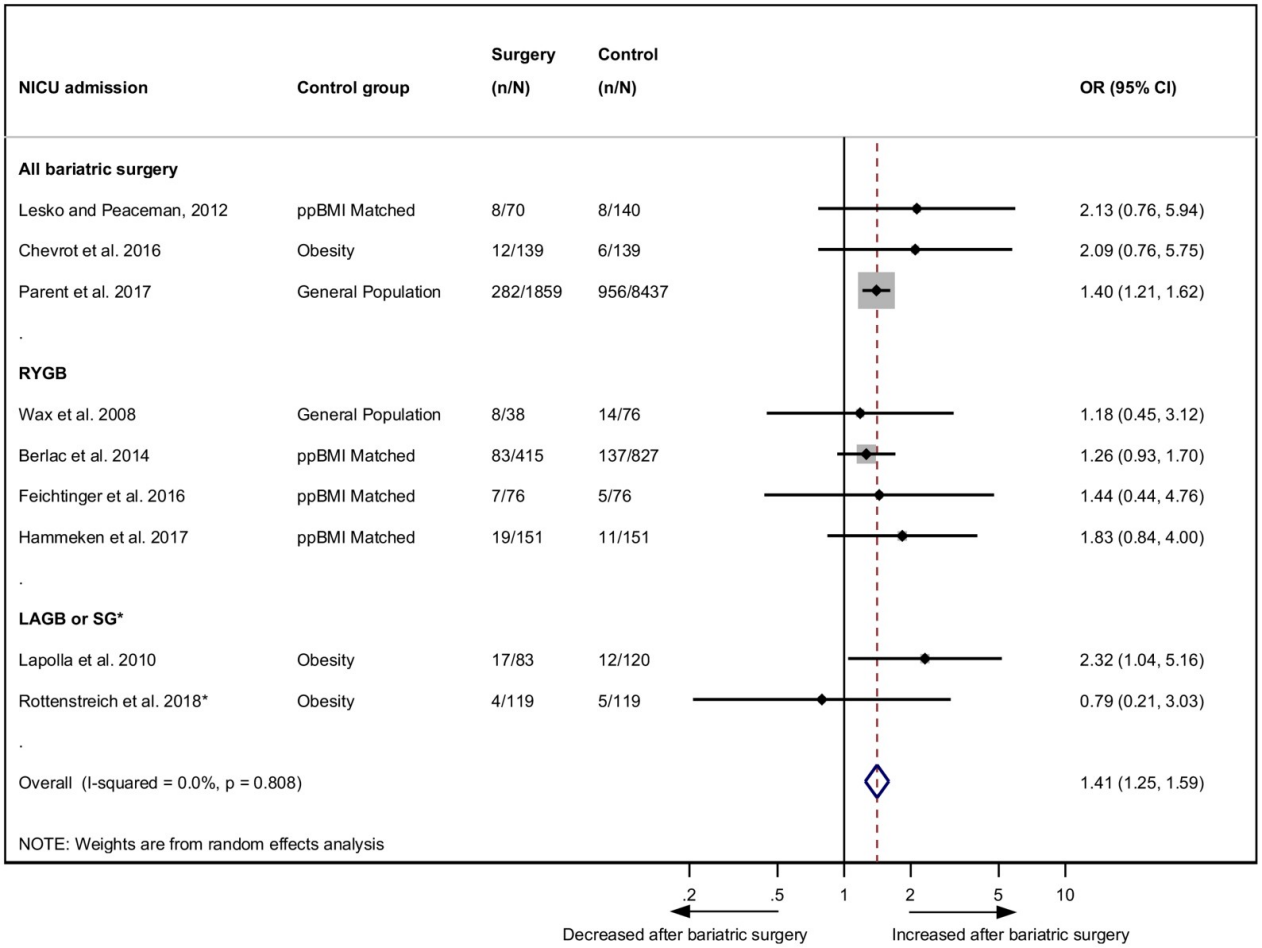
NICU admission after bariatric surgery meta-analysis. Association between maternal bariatric surgery and NICU admission. Studies are presented as Author, year. The forest plot is stratified by type of surgery. *n* = cases of NICU admission. *N* = total group size. *SG only. CI, confidence interval; LAGB, laparoscopic adjustable gastric banding; NICU, neonatal intensive care unit; OR, odds ratio; ppBMI, prepregnancy BMI matched; RYGB, Roux-en-Y gastric bypass; SG, sleeve gastrectomy.

NICU admission was reported in nine studies with babies born post-bariatric surgery being significantly more likely to be admitted to NICU (OR 1.41, 95% CI 1.25–1.59, *p* < 0.001) (Fig 5) [25,27,31,33,38,39,42,46,51]. There was no evidence of heterogeneity (*I*^*2*^ = 0.0%, 95% CI 0.0–64.8, *p* = 0.808).

### Publication bias and sensitivity analyses

There was no evidence of small study effects for any outcome except LGA (*p* = 0.021), which may signal publication bias (S7 Fig, S4 Table). A subset of studies reported both crude and adjusted data for the adverse perinatal outcomes, but when compared, there was little difference in size or direction of associations (S8 Fig). Sensitivity analyses revealed that the results were robust, with only small changes in pooled effect sizes when meta-analysis were repeated with one study excluded (S5 Table).

## Discussion

This systematic review and meta-analysis has demonstrated that perinatal mortality, congenital anomalies, preterm birth, SGA, and NICU admission are associated with increased odds in women who have had bariatric surgery prior to pregnancy compared to women without prior bariatric surgery. Postterm birth and LGA, however, are associated with decreased odds after bariatric surgery. Malabsorptive procedures were associated with a significant increase in SGA and decrease in LGA, whereas restrictive procedures were not. Subgrouping by type of surgery significantly reduced heterogeneity for the outcomes with a high *I*^2^ value, whereas subgrouping by control group did not. There was no evidence of publication bias for any outcome except LGA.

The increase in adverse perinatal outcomes could be related to malnutrition. Unlike restrictive procedures, which reduce stomach size and appetite, malabsorptive procedures bypass a portion of the small intestine where many vitamins and minerals are absorbed, making these patients particularly susceptible to nutrient deficiencies that may negatively affect a subsequent pregnancy [54]. The association between folic acid intake and neural tube defects is well established, and there are links between iron deficiency and preterm birth and between calcium and birth weight [55–57]. Impaired nutrient transport across the placenta is also associated with perinatal morbidity; however, there is limited evidence regarding placental function after bariatric surgery. The studies reporting data on congenital anomalies in pregnancy with and without prior bariatric surgery did not subgroup by type of anomaly—this would be valuable for future research to pinpoint the mechanism behind the anomalies. Another factor that may explain the increase in SGA infants is the increased glycaemic variability and postprandial hypoglycaemia observed after RYGB, as fetal growth has been found to be associated with maternal glucose nadir levels during oral glucose tolerance testing in pregnancy [58].

The strengths of this systematic review and meta-analysis include the thorough search strategy of multiple databases and supplementing this with hand searches of reference lists, citations, and relevant journals. All screening, data extraction, and quality assessment was carried out in duplicate to minimise human error. There are no randomised controlled trials, because of the nature of this research question, but all included studies were medium- to high-quality observational studies. This is the first meta-analysis, to our knowledge, to report significantly increased odds of perinatal mortality and congenital anomalies after bariatric surgery. This is also the first meta-analysis, to our knowledge, to investigate postterm birth after bariatric surgery, for which a significant decrease was found. The results for SGA, LGA, preterm birth, and NICU admission confirm the findings of past meta-analyses but with stronger associations than previously reported and the inclusion of 12 additional studies [9,22,24,27,28,32,33,40,42,46,49,50].

The results from our study are limited by the small sample sizes of some of the included studies. Multiple studies reported few, or even zero, cases of perinatal mortality or congenital anomalies and have therefore resulted in large CIs. Larger epidemiological studies or individual patient data (IPD) meta-analyses need to be carried out for this rare exposure and rare outcome combination. Additionally, there are no large studies exploring congenital anomalies and perinatal mortality specifically after restrictive surgery such as LAGB or SG, which may not have a detrimental effect. A number of studies have reported several adverse perinatal outcomes, many of which are linked, which may result in a loss of statistical and clinical independence. We were unable to include non–English language studies, and one non–English language study meeting our inclusion criteria was excluded. This study from France identified a significant decrease in macrosomia, as our meta-analysis did; however, it also found a decrease in SGA in contrast to the significant increase we found [59].

Women that become pregnant post-bariatric surgery tend to be older than the general population of pregnant women [7]. Many women also still have a BMI > 30 kg/m^2^ despite the weight loss from surgery [45]. There is also evidence that alcohol use and smoking are increased after bariatric surgery [60]. The combination of increased maternal age, high BMI, and unhealthy behaviours in women after bariatric surgery plays a role in the development of adverse perinatal outcomes, in addition to the malnutrition. These are important confounders to consider when investigating perinatal outcomes in this group. When comparing ORs with adjustments made for these factors to unadjusted ORs, we did not see a change in the results. However, in a clinical setting, these factors and behaviours are important for the healthcare provider to take into account because of the evidence of the link with adverse perinatal outcomes. As with all meta-analyses of observational data, unmeasured confounding in the included studies may have implications on the results. Gestational weight gain (GWG) is another factor associated with perinatal outcomes such as birth weight; however, further research is required to determine how the relationship between GWG and pregnancy outcomes differs for women after bariatric surgery and whether current GWG guidelines can apply to this population.

The LAGB subgroup analyses tended to have larger CIs than any other subgroup. This may be due to smaller sample sizes or differences in LAGB band management. Some clinics actively manage gastric bands during pregnancy by deflating in cases of nausea or vomiting and inflating in cases of excess GWG [29]. Future studies should explore how band management could be used to achieve optimal pregnancy outcomes. The studies that combined all types of bariatric surgery drastically differed in surgery type composition, with studies reporting from 13.3% RYGB to 98% RYGB in their cohorts. It would be useful for future studies to separate outcomes by type of surgery or to conduct IPD meta-analyses on the existing data, which would enable standardisation of categories across studies.

Future studies should explore the effect of time to conception after different types of bariatric surgery, especially considering gestational weight loss and advanced maternal age. Many women that are previously considered to be infertile experience increased fertility after bariatric surgery, which may result in unexpected pregnancies immediately after surgery in the rapid weight loss phase [61]. Many clinics recommend waiting 12–18 months to conceive post-surgery, but the evidence base is limited for this.

Bariatric surgery prior to pregnancy is promising for reducing obesity-related comorbidities for the mother, and benefits include reduced risks of gestational diabetes and preeclampsia, which are both serious complications associated with adverse maternal and fetal outcomes. Our meta-analysis has shown that the risks of postterm birth and LGA babies are reduced after bariatric surgery; however, we have also identified adverse outcomes for the baby and efforts now need to be focused on how to reduce these. Internationally, guidelines exist for a variety of high-risk pregnancy groups such as those with diabetes, hypertension, and obesity. This study confirms that bariatric surgery patients that become pregnant are also a high-risk group, and guidelines for health professionals need to be developed as obesity and bariatric surgery increases. The current evidence base could be used to inform risk communication about potential future pregnancies with women of reproductive age prior to surgery. For women with a history of bariatric surgery, preconception nutritional support should be offered, and increased fetal, nutrition, and glucose monitoring is required throughout pregnancy. Further studies are required to determine whether restrictive surgery results in better perinatal outcomes than malabsorptive surgery without compromising maternal outcomes, and if so, these may be the preferred surgery for women of reproductive age.

## Supporting information

S1 Table Search strategy for electronic databases and e-journals. (DOCX)

S2 Table Quality assessment scores for included studies. (DOCX)

S3 Table Meta-regression for outcomes with significant heterogeneity between studies. (DOCX)

S4 Table Eggers test of publication bias for perinatal outcomes after bariatric surgery. (DOCX)

S5 Table Sensitivity analyses for perinatal outcomes after bariatric surgery. (DOCX)

S1 PRISMA Checklist PRISMA checklist for systematic reviews and meta-analyses. (DOC)

S1 Fig Adapted Newcastle-Ottawa quality assessment scale for cohort studies. (DOCX)

S2 Fig Preterm birth after bariatric surgery meta-analysis. (DOCX)

S3 Fig Gestational age (weeks) after bariatric surgery meta-analysis with subtotals by type of surgery. (DOCX)

S4 Fig Small for gestational age after bariatric surgery meta-analysis. (DOCX)

S5 Fig Large for gestational age after bariatric surgery meta-analysis. (DOCX)

S6 Fig Birth weight (grams) after bariatric surgery meta-analysis with subtotals by type of surgery. (DOCX)

S7 Fig Funnel plots of publication bias for perinatal outcomes after bariatric surgery. (DOCX)

S8 Fig Crude versus adjusted data for studies reporting adjusted odds ratios. (DOCX)

## Abbreviations

BMI: body mass index
BPD: biliopancreatic diversion
CI: confidence interval
GWG: gestational weight gain
IPD: individual patient data
LAGB: laparoscopic adjustable gastric banding
LGA: large for gestational age
NICU: neonatal intensive care unit
NR: not reported
OR: odds ratio
RYGB: Roux-en-Y gastric bypass
SG: sleeve gastrectomy
SGA: small for gestational age
WMD: weighted mean difference
