# Supplementary material for: Pregnancy after bariatric surgery and adverse perinatal outcomes: A systematic review and meta-analysis
Source: PLoS Med. 2019 Aug 6;16(8):e1002866. doi: 10.1371/journal.pmed.1002866 (PMC6684044; doi:10.1371/journal.pmed.1002866)
Supplement: S1 Table — (DOCX) [file pmed.1002866.s001.docx]

# S1 Table. Search strategy for electronic databases and e-journals

| **Electronic databases (inception to June 2019)** | |
| --- | --- |
| **Medline and Embase** | 1. exp Pregnancy/ or pregnan$.mp.  2. exp Mother/  3. matern$.mp.  4. (preconception or pre-conception).mp.  5. (pregravid or pre-gravid).mp.  6. (prepregnancy or pre-pregnancy).mp.  7. 1 or 2 or 3 or 4 or 5 or 6  8. exp Bariatric Surgery/ or bariatric surgery.mp.  9. (weight loss surgery or obesity surgery).mp.  10. (gastric bypass or RYGB).mp.  11. (sleeve gastrectomy or gastroplasty or gastric sleeve).mp.  12. (gastric band* or LAGB).mp.  13. (biliopancreatic diversion or BPD or duodenal switch).mp.  14. 8 or 9 or 10 or 11 or 12 or 13  15. exp fetal death/ or exp stillbirth/ or exp perinatal death/  16. exp Fetal Mortality/ or exp Infant Mortality/ or exp Perinatal Mortality/  17. exp Abortion, Spontaneous/  18. (miscarriage or fetal loss).mp.  19. exp "congenital, hereditary, and neonatal diseases and abnormalities"/ or exp congenital abnormalities/ or exp fetal diseases/ or exp genetic diseases, inborn/ or exp infant, newborn, diseases/  20. exp Heart Defects, Congenital/  21. (congenital anomalies or birth defect*).mp.  22. exp Neural Tube Defects/  23. exp Cleft Palate/  24. f*etal.mp.  25. $natal.mp.  26. exp Infant/  27. (newborn* or obstetric* or outcome*).mp. or child/  28. 15 or 16 or 17 or 18 or 19 or 20 or 21 or 22 or 23 or 24 or 25 or 26 or 27  29. 7 and 14 and 28 |
| **PsycINFO** | 1. pregnan$.mp.  2. mother*.mp.  3. matern$.mp.  4. (preconception or pre-conception).mp.  5. (pregravid or pre-gravid).mp.  6. (prepregnancy or pre-pregnancy).mp.  7. 1 or 2 or 3 or 4 or 5 or 6  8. bariatric surgery.mp.  9. (weight loss surgery or obesity surgery).mp.  10. (gastric bypass or RYGB).mp.  11. (sleeve gastrectomy or gastroplasty or gastric sleeve).mp.  12. (gastric band* or LAGB).mp.  13. (biliopancreatic diversion or BPD or duodenal switch).mp. [  14. 8 or 9 or 10 or 11 or 12 or 13  15. (fetal death or stillbirth or perinatal death).mp.  16. (fetal mortality or infant mortality or perinatal mortality).mp.  17. spontaneous abortion.mp.  18. (miscarriage or fetal loss).mp.  19. (congenital abnormalit$ or fetal abnormalit$ or congenital disease* or neonatal disease* or fetal disease*).mp.  20. congenital heart defect*.mp.  21. (congenital anomalies or birth defect*).mp.  22. neural tube defect*.mp.  23. (cleft palate or cleft lip).mp.  24. f*etal.mp.  25. $natal.mp.  26. (infant or child).mp.  27. (newborn* or obstetric* or outcome*).mp.  28. 15 or 16 or 17 or 18 or 19 or 20 or 21 or 22 or 23 or 24 or 25 or 26 or 27  29. 7 and 14 and 28 |
| **CINAHL** | 1. TX pregnan* OR TX mother* OR TX matern*  2. TX bariatric surgery OR TX weight loss surgery OR TX obesity surgery OR TX gastric bypass OR TX gastric band* OR TX sleeve OR TX biliopancreatic diversion OR TX LAGB OR TX RYGB  3. TX death OR TX mortality OR TX newborn* OR TX fetal OR TX congenital OR TX stillbirth OR TX miscarriage OR TX defect* OR TX perinatal OR TX obstetric OR TX neonat* OR TX outcome* OR TX birth  4. 1 and 2 and 3 |
| **Scopus** | (ALL (pregnan* OR mother* OR matern*)) AND (ALL (bariatric surgery OR weight loss AND surgery OR gastric bypass OR gastric band* OR sleeve OR biliopancreatic diversion OR lagb OR rygb)) AND (ALL (death OR mortality OR newborn* OR fetal OR congenital OR stillbirth OR miscarriage OR defect* OR perinatal OR obstetric or neonat* OR outcome* OR birth)) |
| **Google Scholar** | (pregnancy or neonatal or obstetric) AND (outcome) AND (bariatric surgery or bypass or sleeve or biliopancreatic or banding) |
| **Electronic journals (inception to June 2019)** | |
| **Obstetrics and Gynaecology**  **American Journal of Obstetrics and Gynaecology**  **British Journal of Obstetrics and Gynaecology** | bariatric surgery  RYGB  Roux-en-Y  gastric bypass  LAGB  gastric band  biliopancreatic diversion  sleeve gastrectomy |
| **Obesity Surgery**  **Surgery for Obesity and Related Diseases** | pregnancy  neonatal  perinatal  fetal  obstetric  birth  maternal  mother  baby  child |
