# Supplementary material for: Pregnancy after bariatric surgery and adverse perinatal outcomes: A systematic review and meta-analysis
Source: PLoS Med. 2019 Aug 6;16(8):e1002866. doi: 10.1371/journal.pmed.1002866 (PMC6684044; doi:10.1371/journal.pmed.1002866)
Supplement: S2 Table — (DOCX) [file pmed.1002866.s002.docx]

# S2 Table. Quality assessment scores for included studies

| Study  (Author, publication year) | **Newcastle Ottawa Scale Question number and score allocated** | | | | | | | | | **Independent reviewer initials** | |
| --- | --- | --- | --- | --- | --- | --- | --- | --- | --- | --- | --- |
|  | **1** | **2** | **3** | **4** | **5** | **6** | **7** | **Total stars** |  | |  |
| Adams *et al.* 2015 | a* | a* | a* | a* b* | a* | a* | a* | 8 | ZA & LN | |  |
| Belogolovkin *et al.* 2012 | a* | a* | b* | a*b* | a* | a* | b* | 8 | ZA & DC | |  |
| Berglind *et al.* 2014 | a* | a* | a* | b* | a* | a* | c | 6 | ZA & DC | |  |
| Berlac *et al.* 2014 | a* | a* | b* | a* | b* | a* | b* | 7 | ZA & LN | |  |
| Burke *et al.* 2010 | c | a* | b* | a* b* | b* | a* | b* | 7 | ZA & RA | |  |
| Chevrot *et al.* 2016 | a* | a* | a* | a* | a* | a* | a* | 7 | ZA & LN | |  |
| Dell’Agnolo *et al.* 2011 | a* | a* | a* | c | c | a* | b* | 5 | ZA & DC | |  |
| Dixon *et al.* 2005 | a* | a* | a* | a* | a* | a* | b* | 7 | ZA & LN | |  |
| Ducarme *et al.* 2007 | b* | a* | a* | c | a* | a* | a* | 6 | ZA & LN | |  |
| Feichtinger *et al.* 2016 | a* | a* | a* | a* | a* | a* | b* | 7 | ZA & NH | |  |
| Gascoin *et al.* 2017 | a* | a* | a* | a*b* | a* | a* | a* | 8 | ZA & JR | |  |
| Goldman *et al.* 2016 | b* | a* | a* | a* | c | a* | c | 5 | ZA & LN | |  |
| Hammeken *et al.* 2017 | a* | b | b* | a* | a* | a* | b* | 6 | ZA & LN | |  |
| Johansson *et al.* 2015 | a* | a* | b* | a* b* | b* | a* | a* | 8 | ZA & LN | |  |
| Josefsson *et al.* 2013 | a* | a* | b* | a*b* | b* | a* | a* | 8 | ZA & DC | |  |
| Josefsson *et al.* 2011 | a* | a* | b* | c | a* | a* | b* | 6 | ZA & NH | |  |
| Kjaer *et al.* 2013 | a* | a* | b* | a* b* | b* | a* | b* | 8 | ZA & LN | |  |
| Lapolla *et al.* 2010 | a* | a* | a* | c | b* | a* | a* | 6 | ZA & LN | |  |
| Lesko and Peaceman, 2012 | a* | a* | b* | a* b* | b* | a* | b* | 8 | ZA & LN | |  |
| Machado *et al.* 2017 | a* | a* | a* | a* b* | a* | a* | a* | 8 | ZA & LN | |  |
| Marceau *et al.* 2004 | b* | a* | a* | c | c | a* | b* | 5 | ZA & DC | |  |
| Parent *et al.* 2017 | a* | a* | a* | a*b* | b* | a* | a* | 8 | ZA & DC | |  |
| Parker *et al.* 2016 | b* | a* | b* | a* b* | b* | a* | a* | 8 | ZA & DC | |  |
| Patel *et al.* 2008 | a* | a* | a* | c | a* | a* | a* | 6 | ZA & LN | |  |
| Roos *et al.* 2013 | a* | a* | b* | a* b* | b* | a* | a* | 8 | ZA & JR | |  |
| Rottenstreich *et al.* 2018 | b* | a* | a* | a*b* | a* | a* | a* | 8 | ZA & LN | |  |
| Shai *et al.* 2014 | c | a* | b* | a* | a* | a* | a* | 6 | ZA & LN | |  |
| Skull *et al.* 2004 | a* | a* | a* | c | a* | a* | a* | 6 | ZA & DC | |  |
| Stentebjerg *et al.* 2017 | a* | b | a* | c | a* | a* | b* | 5 | ZA & DC | |  |
| Stephansson *et al.* 2018 | b* | a* | a* | a*b* | b* | a* | a* | 8 | ZA & LN | |  |
| Wax *et al.* 2008 | a* | a* | a* | a*b* | a* | a* | a* | 8 | ZA & DC | |  |
| Weintraub *et al.* 2008 | a* | a* | b* | a*b* | a* | a* | b* | 8 | ZA & LN | |  |
| Wittgrove *et al.* 1998 | b* | a* | a* | c | c | a* | b* | 5 | ZA & LN | |  |
| **Total** | a* (n=24)  b* (n=7)  c (n=2) | a* (n=31)  b (n=2) | a* (n=20)  b* (n=13) | a*b* (n=16)  a* (n=7)  b* (n=1)  c (n=9) | a* (n=18)  b* (n=11)  c (n=4) | a* (n=33) | a* (n=17)  b* (n=14)  c (n=2) | 8 (n=15)  7 (n=5)  6 (n=6)  5 (n=5) |  | |  |

Newcastle-Ottawa question numbers 1-7, answers a-d, and associated number of stars (*) are detailed in Figure A2. Minimum number of possible stars to be awarded = 0, maximum number of possible stars to be awarded = 8. Reviewers initials relate to manuscript authors - ZA: Zainab Akhter, JR: Judith Rankin, DC: Dries Ceulemans, LN: Lem Ngongalah, RA: Roger Ackroyd, NH: Nicola Heslehurst.
