# Supplementary material for: Pregnancy after bariatric surgery and adverse perinatal outcomes: A systematic review and meta-analysis
Source: PLoS Med. 2019 Aug 6;16(8):e1002866. doi: 10.1371/journal.pmed.1002866 (PMC6684044; doi:10.1371/journal.pmed.1002866)
Supplement: S3 Table — (DOCX) [file pmed.1002866.s003.docx]

# S3 Table. Meta-regression for outcomes with significant heterogeneity between studies

## S3A Table. Meta-regression for gestational age (weeks)

| Variable | **Number of studies** | **Meta-regression residual *I^2^* (%)** | **Coefficient** | **P value** |
| --- | --- | --- | --- | --- |
| *All studies* | *13* | *66.80* |  |  |
| **Type of surgery**  All bariatric surgery  RYGB or BPD  LAGB | 4  7  2 | 69.37% | -0.0763809 | p=0.58 |
| **Control group**  ppBMI matched  Obesity  General population  Before surgery | 3  3  4  3 | 69.35% | -0.0076803 | p=0.944 |
| **Publication year**  2000–2004  2005–2009  2010–2014  2015+ | 1  4  4  4 | 69.13% | -0.1040644 | p=0.444 |
| **Continent**  Europe  Asia  North America  South America | 6  1  5  1 | 69.15% | 0.0817169 | p=0.488 |
| **Sample size**  1–100  100–1,000  1,000+ | 3  6  4 | 68.34% | -0.0216062 | p=0.901 |
| **Quality score**  5/8  6/8  7/8  8/8 | 2  6  0  5 | 67.88% | 0.1211809 | p=0.241 |

## S3B Table. Meta-regression for large for gestational age

| Variable | **Number of studies** | **Meta-regression residual *I^2^* (%)** | **Coefficient** | **P value** |
| --- | --- | --- | --- | --- |
| *All studies* | *21* | *69.50* |  |  |
| **Type of surgery**  All bariatric surgery  RYGB or BPD  LAGB or SG | 9  8  4 | 67.87 | -0.273883 | p=0.069 |
| **Control group**  ppBMI matched  Obesity  General population  Before surgery | 6  9  3  3 | 70.49 | 0.0391969 | p=0.778 |
| **Large baby definition**  >90^th^ percentile  Mean + 2 S.D or 22%  >4000g | 10  4  7 | 70.85 | 0.0579805 | p=0.734 |
| **Publication year**  1995–1999  2000–2004  2005–2009  2010–2014  2015+ | 1  1  5  6  8 | 67.74 | 0.1626055 | p=0.216 |
| **Continent**  Europe  Asia  North America | 9  3  9 | 68.71 | 0.0109506 | p=0.939 |
| **Sample size**  1–100  100–1,000  1,000–10,000  10,000–100,000  100,000+ | 2  12  3  2  2 | **61.21** | **0.2493393** | **p=0.031** |
| **Quality score**  5/8  6/8  7/8  8/8 | 2  5  4  10 | 68.02 | 0.1293149 | p=0.337 |

Meta-regression was carried for gestational age and large for gestational age (LGA) due to heterogeneity remaining significant after subgroup analyses. Factors that significantly contributed to heterogeneity are highlighted in red. RYGB=Roux-en-Y gastric bypass. BPD=biliopancreatic diversion. LAGB=laparoscopic adjustable gastric banding. SG=sleeve gastrectomy. ppBMI=pre-pregnancy BMI matched. SD=standard deviation.
