# Supplementary material for: Pregnancy after bariatric surgery and adverse perinatal outcomes: A systematic review and meta-analysis
Source: PLoS Med. 2019 Aug 6;16(8):e1002866. doi: 10.1371/journal.pmed.1002866 (PMC6684044; doi:10.1371/journal.pmed.1002866)
Supplement: S4 Table — (DOCX) [file pmed.1002866.s004.docx]

# S4 Table. Eggers test of publication bias for perinatal outcomes after bariatric surgery

| Outcome | **Parameter** | **Estimate** | **Standard error** | **t value** | **p value** | **95% Confidence interval** | |
| --- | --- | --- | --- | --- | --- | --- | --- |
| Perinatal mortality | slope | .3634665 | .2628003 | 1.38 | 0.204 | -.2425521 | .969485 |
|  | bias | -.094103 | .6492512 | -0.14 | 0.888 | -1.591279 | 1.403073 |
|  | Test of H0: no small-study effects P = 0.888, Number of studies = 10, Root MSE = 1.13 | | | | | | |
| Congenital anomalies | slope | .4012084 | .0857801 | 4.68 | 0.002 | .2033991 | .5990176 |
|  | bias | -.6498229 | .4859948 | -1.34 | 0.218 | -1.770529 | .4708832 |
|  | Test of H0: no small-study effects P = 0.218, Number of studies = 10, Root MSE = 1.13 | | | | | | |
| Pre-term birth | slope (coefficient) | .5229677 | .0868996 | 6.02 | 0.000 | .3396256 | .7063099 |
|  | bias (intercept) | -.8105311 | .4509824 | -1.80 | 0.090 | -1.762021 | .1409587 |
|  | Test of H0: no small-study effects P = 0.090, Number of studies = 19, Root MSE = 1.333 | | | | | | |
| Post-term birth | slope | -1.068086 | .1599083 | -6.68 | 0.007 | -1.576986 | -.5591869 |
|  | bias | 1.166715 | .6021039 | 1.94 | 0.148 | -.7494481 | 3.082878 |
|  | Test of H0: no small-study effects P = 0.148, Number of studies = 5, Root MSE = .7966 | | | | | | |
| Small for gestational age | slope | .617479 | .09324 | 6.62 | 0.000 | .4223255 | .8126325 |
|  | bias | .3684037 | .4478583 | 0.82 | 0.421 | -.5689744 | 1.305782 |
|  | Test of H0: no small-study effects P = 0.421, Number of studies = 21, Root MSE = 1.382 | | | | | | |
| Large for gestational age | slope | -.4112801 | .1242798 | -3.31 | 0.004 | -.6714007 | -.1511595 |
|  | bias | -1.372551 | .5464594 | -2.51 | 0.021 | -2.516304 | -.2287987 |
|  | Test of H0: no small-study effects P = 0.021, Number of studies = 21, Root MSE = 1.605 | | | | | | |
| NICU admission | slope | .2979759 | .0669287 | 4.45 | 0.003 | .1397146 | .4562371 |
|  | bias | .3316963 | .3577983 | 0.93 | 0.385 | -.5143622 | 1.177755 |
|  | Test of H0: no small-study effects P = 0.385, Number of studies = 9, Root MSE = .7578 | | | | | | |
| Birth weight (grams) | slope | -314.6102 | 59.62998 | -5.28 | 0.000 | -441.7085 | -187.5119 |
|  | bias | .9364767 | 1.013046 | 0.92 | 0.370 | -1.22278 | 3.095734 |
|  | Test of H0: no small-study effects P = 0.370, Number of studies = 17, Root MSE = 2.037 | | | | | | |
| Gestational age (weeks) | slope | -.1733743 | .2377502 | -0.73 | 0.481 | -.696659 | .3499105 |
|  | bias | .0396638 | 1.136929 | 0.03 | 0.973 | -2.462699 | 2.542027 |
|  | Test of H0: no small-study effects P = 0.973, Number of studies = 13, Root MSE = 1.813 | | | | | | |

Egger's test for small-study effects: Regression of the standard normal deviate of intervention effect estimate against its standard error. Outcomes with significant evidence of publication bias are highlighted in red.
