# Supplementary material for: Pregnancy after bariatric surgery and adverse perinatal outcomes: A systematic review and meta-analysis
Source: PLoS Med. 2019 Aug 6;16(8):e1002866. doi: 10.1371/journal.pmed.1002866 (PMC6684044; doi:10.1371/journal.pmed.1002866)
Supplement: S5 Table — (DOCX) [file pmed.1002866.s005.docx]

# S5 Table. Sensitivity analyses for perinatal outcomes after bariatric surgery

| Perinatal mortality | Study excluded | **Odds ratio (95% CI)** | **Interpretation** | ***I^2^* statistic (%)** |
| --- | --- | --- | --- | --- |
|  | All studies included (before sensitivity analysis) | 1.38 (1.03–1.85) | Significant increase | 12.1 |
|  | Weintraub *et al.* 2008 | 1.48 (1.14–1.91) | Strongest association | 0.0 |
|  | Burke *et al.* 2010 | 1.34 (0.99–1.82) | Change in significance | 14.6 |
|  | Lesko and Peaceman, 2012 | 1.37 (1.06–1.76) |  | 0.0 |
|  | Roos *et al.* 2013 | 1.38 (0.94–2.03) | Change in significance | 21.4 |
|  | Johansson *et al.* 2015 | 1.33 (0.96–1.84) | Change in significance | 17.6 |
|  | Parent *et al.* 2017 | 1.32 (0.89–1.95) | Weakest association  Change in significance | 20.2 |
|  | Marceau *et al.* 2004 (BPD) | 1.40 (1.03–1.90) |  | 17.1 |
|  | Berlac *et al.* 2014 | 1.39 (1.01–1.91) |  | 20.7 |
|  | Adams *et al.* 2015 | 1.40 (1.03–1.89) |  | 16.1 |
|  | Parker *et al.* 2016 | 1.34 (0.91–1.96) | Change in significance | 21.5 |
| Congenital anomalies | Study excluded | **Odds ratio (95% CI)** | **Interpretation** | ***I^2^* statistic (%)** |
|  | All studies included (before sensitivity analysis) | 1.29 (1.04–1.59) | Significant increase | 28.0 |
|  | Weintraub *et al.* 2008 | 1.25 (1.03–1.52) |  | 18.3 |
|  | Josefsson *et al.* 2013 | 1.28 (1.00–1.64) |  | 34.9 |
|  | Johansson *et al.* 2015 | 1.42 (1.27–1.59) | Strongest association | 0.0 |
|  | Parent *et al.* 2017 | 1.21 (0.91–1.60) | Weakest association  Change in significance | 24.4 |
|  | Marceau *et al.* 2004 (BPD) | 1.26 (1.00–1.59) |  | 35.3 |
|  | Patel *et al.* 2008 | 1.29 (1.03–1.60) |  | 33.7 |
|  | Wax *et al.* 2008 | 1.30 (1.06–1.60) |  | 26.7 |
|  | Berlac *et al.* 2014 | 1.30 (1.01–1.66) |  | 33.0 |
|  | Adams *et al.* 2015 | 1.27 (0.98–1.64) | Change in significance | 35.7 |
|  | Rottenstreich *et al.* 2018 | 1.28 (1.02–1.61) |  | 35.5 |
| Pre–term birth | Study excluded | **Odds ratio (95% CI)** | **Interpretation** | ***I^2^* statistic (%)** |
|  | All bariatric surgery (before sensitivity analysis) | 1.57 (1.38–1.79) | Significant increase | 21.0 |
|  | Dell'Agnolo *et al.* 2011 | 1.57 (1.37–1.79) |  | 27.4 |
|  | Josefsson *et al.* 2011 | 1.56 (1.36–1.80) |  | 30.0 |
|  | Lesko and Peaceman, 2012 | 1.60 (1.46–1.76) |  | 0.0 |
|  | Kjaer *et al.* 2013 | 1.59 (1.39–1.82) |  | 24.1 |
|  | Roos *et al.* 2013 | 1.50 (1.24–1.81) |  | 29.4 |
|  | Shai *et al.* 2014 | 1.64 (1.49–1.81) | Strongest association | 1.4 |
|  | Johansson *et al.* 2015 | 1.60 (1.40–1.84) |  | 21.4 |
|  | Chevrot *et al.* 2016 | 1.57 (1.37–1.80) |  | 28.0 |
|  | Parent *et al.* 2017 | 1.49 (1.26–1.75) | Weakest association | 18.4 |
|  | RYGB (before sensitivity analysis) | 1.14 (0.89–1.46) | Non-significant result | 9.8 |
|  | Wittgrove *et al.* 1998 | 1.19 (0.90–1.57) |  | 21.5 |
|  | Patel *et al.* 2008 | 1.16 (0.87–1.55) |  | 20.9 |
|  | Wax *et al.* 2008 | 1.18 (0.87–1.58) |  | 22.0 |
|  | Kjaer *et al.* 2013 | 1.15 (0.83–1.59) |  | 16.8 |
|  | Adams *et al.* 2015 | 1.41 (1.04–1.93) | Strongest association  Change in significance | 0.0 |
|  | Chevrot *et al.* 2016 | 1.19 (0.88–1.59) |  | 22.7 |
|  | Goldman *et al.* 2016 | 1.18 (0.89–1.56) |  | 22.6 |
|  | Stentebjerg *et al.* 2017 | 1.01 (0.81–1.27) | Weakest association | 0.0 |
|  | LAGB and*/or^†^ SG (before sensitivity analysis) | 0.88 (0.58–1.34) | Non-significant result | 0.0 |
|  | Dixon *et al.* 2005 | 0.98 (0.63–1.54) | Weakest association | 0.0 |
|  | Ducarme *et al.* 2007 | 0.88 (0.57–1.34) |  | 0.0 |
|  | Lapolla *et al.* 2010 | 0.72 (0.44–1.19) | Strongest association | 0.0 |
|  | Kjaer *et al.* 2013 | 0.82 (0.53–1.29) |  | 0.0 |
|  | Chevrot *et al.* 2016* | 0.91 (0.58–1.44) |  | 0.0 |
|  | Goldman *et al.* 2016 | 0.90 (0.59–1.38) |  | 0.0 |
|  | Rottenstreich *et al.* 2018^†^ | 0.98 (0.62–1.57) | Weakest association | 0.0 |
| Post–term birth | Study excluded | **Odds ratio (95% CI)** | **Interpretation** | ***I^2^* statistic (%)** |
|  | All studies included (before sensitivity analysis) | 0.46 (0.35–0.60) | Significant decrease | 7.2 |
|  | Kjaer *et al.* 2013 | 0.41 (0.32–0.53) | Strongest association | 0.0 |
|  | Stephansson *et al.* 2018 | 0.63 (0.41–0.96) | Weakest association | 0.0 |
|  | Patel *et al.* 2008 | 0.46 (0.34–0.62) |  | 15.9 |
|  | Wax *et al.* 2008 | 0.49 (0.34–0.70) |  | 30.3 |
|  | Adams *et al.* 2015 | 0.47 (0.32–0.70) |  | 21.1 |
| Small for gestational age | Study excluded | **Odds ratio (95% CI)** | **Interpretation** | ***I^2^* statistic (%)** |
|  | All bariatric surgery (before sensitivity analysis) | 1.87 (1.61–2.17) | Significant increase | 23.6 |
|  | Weintraub *et al.* 2008 | 1.90 (1.61–2.23) |  | 33.1 |
|  | Dell'Agnolo *et al.* 2011 | 1.88 (1.61–2.20) |  | 31.4 |
|  | Josefsson *et al.* 2011 | 1.84 (1.59–2.13) |  | 24.0 |
|  | Lesko and Peaceman, 2012 | 1.88 (1.60–2.20) |  | 31.4 |
|  | Kjaer *et al.* 2013 | 1.81 (1.57–2.08) |  | 18.1 |
|  | Roos *et al.* 2013 | 1.98 (1.60–2.44) |  | 33.1 |
|  | Johansson *et al.* 2015 | 1.70 (1.51–1.91) | Weakest association | 0.6 |
|  | Chevrot *et al.* 2016 | 1.87 (1.60–2.20) |  | 30.8 |
|  | Parent *et al.* 2017 | 2.02 (1.75–2.34) | Strongest association | 0.0 |
|  | RYGB (before sensitivity analysis) | 2.72 (2.32–3.20) | Significant increase | 0.0 |
|  | Marceau *et al.* 2004* | 2.69 (2.28–3.18) |  | 0.0 |
|  | Patel *et al.* 2008 | 2.71 (2.31–3.19) |  | 0.0 |
|  | Wax *et al.* 2008 | 2.76 (2.35–3.25) |  | 0.0 |
|  | Kjaer *et al.* 2013 | 2.71 (2.29–3.20) |  | 0.0 |
|  | Adams *et al.* 2015 | 2.83 (2.37–3.38) | Strongest association | 0.0 |
|  | Chevrot *et al.* 2016 | 2.64 (2.24–3.11) | Weakest association | 0.0 |
|  | Feichtinger *et al.* 2016 | 2.71 (2.30–3.19) |  | 0.0 |
|  | Parker *et al.* 2016 | 2.78 (2.18–3.54) |  | 0.1 |
|  | Hammeken *et al.* 2017 | 2.71 (2.31–3.21) |  | 0.5 |
|  | Machado *et al.* 2017 | 2.71 (2.31–3.19) |  | 0.0 |
|  | LAGB and^†^/or^‡^ SG (before sensitivity analysis) | 1.25 (0.62–2.51) | Non-significant result | 34.0 |
|  | Dixon *et al.* 2005 | 1.45 (0.66–3.19) |  | 33.2 |
|  | Ducarme *et al.* 2007 | 1.30 (0.59–2.85) |  | 43.9 |
|  | Lapolla *et al.* 2010 | 1.47 (0.75–2.86) | Strongest association | 25.6 |
|  | Kjaer *et al.* 2013 | 1.22 (0.53–2.83) |  | 46.7 |
|  | Chevrot *et al.* 2016^†^ | 1.10 (0.44–2.78) |  | 46.7 |
|  | Rottenstreich *et al.* 2018^‡^ | 0.93 (0.49–1.77) | Weakest association | 0.0 |
| Large for gestational age | Study excluded | **Odds ratio (95% CI)** | **Interpretation** | ***I^2^* statistic (%)** |
|  | All bariatric surgery (before sensitivity analysis) | 0.51 (0.39–0.67) | Significant decrease | 70.9 |
|  | Weintraub *et al.* 2008 | 0.52 (0.40–0.69) |  | 73.4 |
|  | Burke *et al.* 2010 | 0.48 (0.36–0.65) |  | 72.7 |
|  | Josefsson *et al.* 2011 | 0.49 (0.38–0.65) |  | 73.2 |
|  | Lesko and Peaceman, 2012 | 0.52 (0.40–0.68) |  | 73.6 |
|  | Kjaer *et al.* 2013 | 0.53 (0.41–0.70) |  | 71.7 |
|  | Roos *et al.* 2013 | 0.50 (0.35–0.70) |  | 76.4 |
|  | Johansson *et al.* 2015 | 0.58 (0.47–0.72) | Weakest association | 46.1 |
|  | Chevrot *et al.* 2016 | 0.52 (0.39–0.69) |  | 74.0 |
|  | Parent *et al.* 2017 | 0.47 (0.36–0.62) | Strongest association | 59.1 |
|  | RYGB or BPD* (before sensitivity analysis) | 0.24 (0.14–0.41) | Significant decrease | 70.1 |
|  | Wittgrove *et al.* 1998 | 0.25 (0.14–0.44) |  | 71.8 |
|  | Marceau *et al.* 2004* | 0.28 (0.16–0.47) | Weakest association | 58.5 |
|  | Patel *et al.* 2008 | 0.24 (0.14–0.42) |  | 72.9 |
|  | Wax *et al.* 2008 | 0.23 (0.13–0.41) |  | 73.4 |
|  | Kjaer *et al.* 2013 | 0.27 (0.16–0.46) |  | 68.4 |
|  | Adams *et al.* 2015 | 0.21 (0.10–0.44) | Strongest association | 71.1 |
|  | Chevrot *et al.* 2016 | 0.23 (0.13–0.42) |  | 73.0 |
|  | Feichtinger *et al.* 2016 | 0.24 (0.14–0.42) |  | 73.0 |
|  | Parker *et al.* 2016 | 0.23 (0.17–0.31) |  | 0.0 |
|  | Hammeken *et al.* 2017 | 0.25 (0.14–0.43) |  | 72.5 |
|  | LAGB and^†^/or^‡^ SG (before sensitivity analysis) | 0.59 (0.30–1.14) | Non-significant result | 61.8 |
|  | Dixon *et al.* 2005 | 0.56 (0.24–1.32) |  | 69.6 |
|  | Ducarme *et al.* 2007 | 0.59 (0.28–1.23) |  | 69.3 |
|  | Lapolla *et al.* 2010 | 0.55 (0.22–1.37) |  | 69.8 |
|  | Kjaer *et al.* 2013 | 0.48 (0.24–0.95) | Strongest association  Change in significance | 56.2 |
|  | Chevrot *et al.* 2016^†^ | 0.52 (0.22–1.23) |  | 68.3 |
|  | Rottenstreich *et al.* 2018^‡^ | 0.77 (0.52–1.14) | Weakest association | 0.0 |
| NICU admission | Study excluded | **Odds ratio (95% CI)** | **Interpretation** | ***I^2^* statistic (%)** |
|  | All studies included (before sensitivity analysis) | 1.41 (1.25–1.59) | Significant increase | 0.0 |
|  | Lesko and Peaceman, 2012 | 1.40 (1.24–1.58) |  | 0.0 |
|  | Chevrot *et al.* 2016 | 1.40 (1.24–1.58) |  | 0.0 |
|  | Parent *et al.* 2017 | 1.43 (1.13–1.81) | Strongest association | 0.0 |
|  | Wax *et al.* 2008 | 1.41 (1.25–1.60) |  | 0.0 |
|  | Berlac *et al.* 2014 | 1.44 (1.26–1.64) |  | 0.0 |
|  | Feichtinger *et al.* 2016 | 1.41 (1.24–1.59) |  | 0.0 |
|  | Hammeken *et al.* 2017 | 1.40 (1.24–1.58) |  | 0.0 |
|  | Lapolla *et al.* 2010 | 1.39 (1.23–1.57) | Weakest association | 0.0 |
|  | Rottenstreich *et al.* 2018 | 1.41 (1.25–1.60) |  | 0.0 |
| Birth weight | Study excluded | **Weighted mean difference in grams (95% CI)** | **Interpretation** | ***I^2^* statistic (%)** |
|  | All bariatric surgery (before sensitivity analysis) | -223.71 (-273.68, -173.74) | Significant decrease | 21.2 |
|  | Weintraub *et al.* 2008 | -238.01 (-294.83, -181.20) | Strongest association | 16.1 |
|  | Josefsson *et al.* 2011 | -233.36 (-286.85, -179.86) |  | 21.9 |
|  | Belogolovkin *et al.* 2012 | -188.05 (-244.64, -131.46) | Weakest association | 0.0 |
|  | Berglind *et al.* 2014 | -222.38 (-281.83, -162.93) |  | 39.2 |
|  | Chevrot *et al.* 2016 | -221.12 (-284.20, -158.05) |  | 39.6 |
|  | RYGB (before sensitivity analysis) | -226.10 (-273.43, -178.78) | Significant decrease | 0.0 |
|  | Patel *et al.* 2008 | -230.01 (-279.76, -180.26) |  | 2.7 |
|  | Wax *et al.* 2008 | -230.91 (-285.82, -176.00) |  | 9.0 |
|  | Adams *et al.* 2015 | -258.25 (-328.95, -187.55) | Strongest association | 0.0 |
|  | Goldman *et al.* 2016 | -220.70 (-268.37, -173.03) |  | 0.0 |
|  | Gascoin *et al.* 2017 | -217.62 (-266.54, -168.71) | Weakest association | 0.0 |
|  | Hammeken *et al.* 2017 | -223.32 (-277.72, -168.92) |  | 3.7 |
|  | Machado *et al.* 2017 | -234.64 (-291.80, -177.48) |  | 8.5 |
|  | Stentebjerg *et al.* 2017 | -234.80 (-294.66, -174.94) |  | 9.2 |
| Gestational age | Study excluded | **Weighted mean difference in weeks (95% CI)** | **Interpretation** | ***I^2^* statistic (%)** |
|  | All bariatric surgery (before sensitivity analysis) | -0.11 (-0.57, 0.36) | Non-significant result | 83.8 |
|  | Weintraub *et al.* 2008 | -0.21 (-0.78, 0.35) |  | 82.5 |
|  | Josefsson *et al.* 2011 | 0.01 (-0.57, 0.59) | Weakest association | 88.3 |
|  | Belogolovkin *et al.* 2012 | 0.05 (-0.42, 0.53) |  | 71.0 |
|  | Berglind *et al.* 2014 | -0.26 (-0.75, 0.23) | Strongest association | 82.1 |
|  | RYGB or BPD* (before sensitivity analysis) | -0.24 (-0.55, 0.06) | Non-significant result | 63.6 |
|  | Marceau *et al.* 2004* | -0.28 (-0.69, 0.14) |  | 69.3 |
|  | Patel *et al.* 2008 | -0.24 (-0.56, 0.08) |  | 69.5 |
|  | Wax *et al.* 2008 | -0.25 (-0.58, 0.08) |  | 69.7 |
|  | Adams *et al.* 2015 | -0.34 (-0.70, 0.03) |  | 59.9 |
|  | Hammeken *et al.* 2017 | -0.17 (-0.50, 0.15) |  | 61.8 |
|  | Machado *et al.* 2017 | -0.34 (-0.67, -0.01) | Strongest association  Change in significance | 62.6 |
|  | Stentebjerg *et al.* 2017 | -0.11 (-0.35, 0.13) | Weakest association | 38.7 |

Sensitivity analyses were performed by excluding one study at a time from the meta-analyses to identify the effect of any one study on the pooled effect size and between-study heterogeneity (*I^2^* statistic). The ‘Interpretation’ column outlines when the removal of a study resulted in: lost or gained statistical significance, the weakest association (odds ratio closer to 1 or weighted mean difference closest to 0), or the strongest association (odds ratio furthest from 1 or weighted mean difference furthest from 0) for each outcome. CI=confidence interval. RYGB=Roux-en-Y gastric bypass. BPD=biliopancreatic diversion. LAGB=laparoscopic adjustable gastric banding. SG=sleeve gastrectomy. NICU=neonatal intensive care unit.
