# Supplementary material for: Pregnancy after bariatric surgery and adverse perinatal outcomes: A systematic review and meta-analysis
Source: PLoS Med. 2019 Aug 6;16(8):e1002866. doi: 10.1371/journal.pmed.1002866 (PMC6684044; doi:10.1371/journal.pmed.1002866)
Supplement: S1 Fig — (DOCX) [file pmed.1002866.s007.docx]

# S1 Figure. Adapted Newcastle-Ottawa quality assessment scale for cohort studies

Study – author and year:

Reviewer initials:

**Selection (exposure = bariatric surgery prior to pregnancy)**

1) Representativeness of the exposed cohort

a) truly representative of the average post-bariatric surgery pregnant woman in the community **★**

(A random sample of OR all of the women who became pregnant after bariatric surgery in a defined area during a defined time period)

b) somewhat representative of the average post-bariatric surgery pregnant woman in the community **★**

c) selected group of users

(e.g. Those with diabetes, private UK hospital patients only)

d) no description of the derivation of the cohort

2) Selection of the non-exposed cohort

a) drawn from the same community as the exposed cohort **★**

(From the same hospital/maternity unit or perinatal database)

b) drawn from a different source

c) no description of the derivation of the non-exposed cohort

3) Ascertainment of exposure

a) medical/hospital records **★**

b) database (ICD codes) **★**

c) self-report

d) no description

**Comparability**

4) Comparability of cohorts on the basis of the design or analysis

a) study controls for maternal age **★**

b) study controls for any additional factor (pre-gestational DM or socioeconomic status/lifestyle factors) **★**

**Outcome (perinatal outcome)**

5) Assessment of outcome

a) medical/hospital records **★**

b) database (ICD codes) **★**

c) self-report

d) no description

6) Was follow-up long enough for outcomes to occur

a) yes (women were followed until the end of pregnancy) **★**

b) no

7) Adequacy of follow up of cohorts

a) complete follow up - all subjects accounted for **★**

b) subjects lost to follow up unlikely to introduce bias, small number lost, >80% or description provided of those lost **★**

c) follow up rate < 80% and no description of those lost

d) no statement

**Total number of stars:** /8

Notes:

The Newcastle-Ottawa quality assessment scale was adapted to meet the needs of this research question (changes highlighted in red).

A study can be awarded a maximum of one star for each numbered item within the Selection and Outcome categories. A maximum of two stars can be given for Comparability.

The question “Demonstration that outcome of interest was not present at start of study” was not applicable to this review as an adverse perinatal outcome cannot occur before a pregnancy and was therefore removed.
