# Supplementary material for: Pregnancy after bariatric surgery and adverse perinatal outcomes: A systematic review and meta-analysis
Source: PLoS Med. 2019 Aug 6;16(8):e1002866. doi: 10.1371/journal.pmed.1002866 (PMC6684044; doi:10.1371/journal.pmed.1002866)
Supplement: S2 Fig — (DOCX) [file pmed.1002866.s008.docx]

# S2 Figure: Pre-term birth after bariatric surgery meta-analysis

## S2A Figure: Pre-term birth meta-analysis with all studies together

NOTE: Weights are from random effects analysis

Overall (I-squared = 50.1%, p = 0.007)

Shai et al. 2014

Chevrot et al. 2016

Lapolla et al. 2010

**Pre-term birth**

Stentebjerg et al. 2017

Lesko and Peaceman, 2012

Rottenstreich et al. 2018

Roos et al. 2013

Patel et al. 2008

Goldman et al. 2016

Kjaer et al. 2013

Ducarme et al. 2017

Adams et al. 2015

Josefsson et al. 2011

Dell'Agnolo et al. 2011

Wax et al. 2008

Johansson et al. 2015

Dixon et al. 2005

Wittgrove et al. 1998

Parent et al. 2017

1.35 (1.14, 1.60)

1.09 (0.69, 1.72)

1.22 (0.51, 2.92)

1.43 (0.67, 3.09)

**OR (95% CI)**

2.28 (1.17, 4.46)

3.64 (1.49, 8.90)

0.59 (0.23, 1.47)

1.66 (1.43, 1.93)

1.37 (0.48, 3.90)

1.20 (0.14, 10.12)

1.31 (0.85, 2.04)

1.11 (0.14, 8.81)

0.87 (0.65, 1.17)

1.45 (0.78, 2.69)

0.67 (0.06, 7.97)

1.24 (0.50, 3.05)

1.37 (1.00, 1.87)

0.47 (0.15, 1.43)

0.83 (0.17, 4.12)

1.74 (1.50, 2.03)

813/7242

24/326

12/139

15/83

**Surgery**

**(n/N)**

10/71

14/70

8/119

243/2511

7/26

2/12

29/339

1/13

96/764

11/126

2/41

10/38

59/590

5/79

4/36

261/1859

17627/274487

110/1612

10/139

16/120

**Control**

**(n/N)**

3884/57970

9/140

13/119

750/12379

14/66

2/14

85/1277

29/414

108/764

11667/188500

1/14

17/76

176/2344

10/79

3/23

723/8437

100.00

7.61

3.13

3.84

**%**

**Weight**

4.71

3.01

2.88

14.75

2.31

0.62

7.96

0.66

11.16

5.26

0.46

2.97

10.80

2.05

1.08

14.76

1.35 (1.14, 1.60)

1.09 (0.69, 1.72)

1.22 (0.51, 2.92)

1.43 (0.67, 3.09)

2.28 (1.17, 4.46)

3.64 (1.49, 8.90)

0.59 (0.23, 1.47)

1.66 (1.43, 1.93)

1.37 (0.48, 3.90)

1.20 (0.14, 10.12)

1.31 (0.85, 2.04)

1.11 (0.14, 8.81)

0.87 (0.65, 1.17)

1.45 (0.78, 2.69)

0.67 (0.06, 7.97)

1.24 (0.50, 3.05)

1.37 (1.00, 1.87)

0.47 (0.15, 1.43)

0.83 (0.17, 4.12)

1.74 (1.50, 2.03)

813/7242

24/326

12/139

15/83

10/71

14/70

8/119

243/2511

7/26

2/12

29/339

1/13

96/764

11/126

2/41

10/38

59/590

5/79

4/36

261/1859

1

.2

.5

1

2

5

10

Decreased after bariatric surgery Increased after bariatric surgery

Association between maternal bariatric surgery and pre-term birth. Studies are presented as: Author, year. OR=odds ratio. CI=confidence interval. n=cases of pre-term birth. N=total group size.

## S2B Figure: Pre-term birth meta-analysis with subtotals by control group

NOTE: Weights are from random effects analysis

.

.

.

.

.

**ppBMI Matched**

Lesko and Peaceman, 2012 (All)

Kjaer et al. 2013 (RYGB)

Kjaer et al. 2013 (LAGB)

Roos et al. 2013 (All)

Adams et al. 2015 (RYGB)

Chevrot et al. 2016 (LAGB & SG)

Chevrot et al. 2016 (RYGB)

Subtotal (I-squared = 70.8%, p = 0.002)

**Obesity**

Dixon et al. 2005 (LAGB)

Ducarme et al. 2007 (LAGB)

Patel et al. 2008 (RYGB)

Lapolla et al. 2010 (LAGB)

Lesko and Peaceman, 2012 (All)

Shai et al. 2014 (All)

Johansson et al. 2015 (All)

Chevrot et al. 2016 (All)

Goldman et al. 2016 (LAGB)

Goldman et al. 2016 (RYGB)

Rottenstreich et al. 2018 (SG)

Subtotal (I-squared = 0.0%, p = 0.675)

**Healthy BMI**

Patel et al. 2008 (RYGB)

Lapolla et al. 2010 (LAGB)

Subtotal (I-squared = 83.1%, p = 0.015)

**General Population**

Dixon et al. 2005 (LAGB)

Wax et al. 2008 (RYGB)

Josefsson et al. 2011 (All)

Roos et al. 2013 (All)

Parent et al. 2017 (All)

Stentebjerg et al. 2017 (RYGB)

Subtotal (I-squared = 11.2%, p = 0.344)

**Before Surgery**

Wittgrove et al. 1998 (RYGB)

Lapolla et al. 2010 (LAGB)

Dell'Agnolo et al. 2011 (All)

Adams et al. 2015 (RYGB)

Goldman et al. 2016 (LAGB)

Goldman et al. 2016 (RYGB)

Subtotal (I-squared = 0.0%, p = 0.584)

**Pre-term birth**

3.64 (1.49, 8.90)

1.29 (0.80, 2.07)

1.45 (0.44, 4.76)

1.66 (1.43, 1.93)

0.87 (0.65, 1.17)

0.78 (0.28, 2.13)

1.12 (0.40, 3.10)

1.33 (0.93, 1.90)

0.47 (0.15, 1.43)

1.11 (0.14, 8.81)

1.37 (0.48, 3.90)

1.43 (0.67, 3.09)

1.69 (0.79, 3.65)

1.09 (0.69, 1.72)

1.37 (1.00, 1.87)

1.22 (0.51, 2.92)

0.46 (0.04, 5.77)

1.20 (0.14, 10.12)

0.59 (0.23, 1.47)

1.21 (0.98, 1.49)

1.45 (0.57, 3.71)

5.88 (3.03, 11.43)

3.04 (0.75, 12.29)

0.91 (0.37, 2.26)

1.24 (0.50, 3.05)

1.45 (0.78, 2.69)

1.97 (1.73, 2.25)

1.74 (1.50, 2.03)

2.28 (1.17, 4.46)

1.83 (1.62, 2.05)

0.83 (0.17, 4.12)

1.00 (0.13, 7.67)

0.67 (0.06, 7.97)

2.34 (2.02, 2.71)

1.00 (0.08, 12.07)

3.40 (0.42, 27.29)

2.30 (1.99, 2.65)

**OR (95% CI)**

14/70

25/286

4/53

243/2511

96/764

6/81

6/58

394/3823

5/79

1/13

7/26

15/83

14/70

24/326

59/590

12/139

1/14

2/12

8/119

148/1471

7/26

15/83

22/109

5/79

10/38

11/126

243/2511

261/1859

10/71

540/4684

4/36

2/27

2/41

307/2666

1/14

2/12

318/2796

**Surgery**

9/140

74/1070

11/207

750/12379

108/764

13/139

13/139

978/14838

10/79

29/414

14/66

16/120

18/140

110/1612

176/2344

10/139

2/14

2/14

13/119

400/5061

38/188

31/858

69/1046

4209/61000

17/76

11667/188500

86830/1686830

723/8437

3884/57970

107330/2002813

3/23

2/27

1/14

551/10447

2/28

2/36

561/10575

**(n/N)**

**Control**

9.94

18.19

6.72

25.64

22.68

8.48

8.35

100.00

3.39

0.99

3.89

7.25

7.28

20.34

44.58

5.60

0.67

0.94

5.05

100.00

47.21

52.79

100.00

1.65

1.67

3.49

49.05

41.14

3.00

100.00

0.82

0.51

0.34

97.51

0.34

0.48

100.00

**Weight**

**%**

3.64 (1.49, 8.90)

1.29 (0.80, 2.07)

1.45 (0.44, 4.76)

1.66 (1.43, 1.93)

0.87 (0.65, 1.17)

0.78 (0.28, 2.13)

1.12 (0.40, 3.10)

1.33 (0.93, 1.90)

0.47 (0.15, 1.43)

1.11 (0.14, 8.81)

1.37 (0.48, 3.90)

1.43 (0.67, 3.09)

1.69 (0.79, 3.65)

1.09 (0.69, 1.72)

1.37 (1.00, 1.87)

1.22 (0.51, 2.92)

0.46 (0.04, 5.77)

1.20 (0.14, 10.12)

0.59 (0.23, 1.47)

1.21 (0.98, 1.49)

1.45 (0.57, 3.71)

5.88 (3.03, 11.43)

3.04 (0.75, 12.29)

0.91 (0.37, 2.26)

1.24 (0.50, 3.05)

1.45 (0.78, 2.69)

1.97 (1.73, 2.25)

1.74 (1.50, 2.03)

2.28 (1.17, 4.46)

1.83 (1.62, 2.05)

0.83 (0.17, 4.12)

1.00 (0.13, 7.67)

0.67 (0.06, 7.97)

2.34 (2.02, 2.71)

1.00 (0.08, 12.07)

3.40 (0.42, 27.29)

2.30 (1.99, 2.65)

14/70

25/286

4/53

243/2511

96/764

6/81

6/58

394/3823

5/79

1/13

7/26

15/83

14/70

24/326

59/590

12/139

1/14

2/12

8/119

148/1471

7/26

15/83

22/109

5/79

10/38

11/126

243/2511

261/1859

10/71

540/4684

4/36

2/27

2/41

307/2666

1/14

2/12

318/2796

**(n/N)**

1

.1

.5

1

2

5

10

15

Decreased after bariatric surgery Increased after bariatric surgery

Association between maternal bariatric surgery and pre-term birth. Studies are presented as: Author, year (type of bariatric surgery). Results are subgrouped by control group. n=cases of pre-term birth. N=total group size. OR=odds ratio. CI=confidence interval. ppBMI=pre-pregnancy body mass index. All=all bariatric surgery. RYGB=Roux-en-Y gastric bypass. LAGB=laparoscopic adjustable gastric banding. SG=sleeve gastrectomy.
