# Supplementary material for: Pregnancy after bariatric surgery and adverse perinatal outcomes: A systematic review and meta-analysis
Source: PLoS Med. 2019 Aug 6;16(8):e1002866. doi: 10.1371/journal.pmed.1002866 (PMC6684044; doi:10.1371/journal.pmed.1002866)
Supplement: S3 Fig — (DOCX) [file pmed.1002866.s009.docx]

# S3 Figure: Gestational age (weeks) after bariatric surgery meta-analysis with subtotals by type of surgery

NOTE: Weights are from random effects analysis

.

.

.

Overall (I-squared = 66.8%, p = 0.000)

**RYGB (or BPD*)**

Ducarme et al. 2007

Subtotal (I-squared = 0.0%, p = 0.737)

Patel et al. 2008

**Gestational age (weeks)**

Adams et al. 2015

Machado et al. 2017

Hammeken et al. 2017

Weintraub et al. 2008

Stentebjerg et al. 2017

Marceau et al. 2004*

**All bariatric surgery**

Josefsson et al. 2011

Lapolla et al. 2010

Berglind et al. 2014

Belogolovkin et al. 2012

Subtotal (I-squared = 83.8%, p = 0.000)

Subtotal (I-squared = 63.6%, p = 0.011)

Wax et al. 2008

**LAGB**

-0.16 (-0.38, 0.06)

0.20 (-0.64, 1.04)

0.10 (-0.49, 0.68)

-0.40 (-1.77, 0.97)

WMD (95% CI)

0.07 (-0.20, 0.34)

0.23 (-0.22, 0.68)

-0.60 (-1.10, -0.10)

0.20 (-0.15, 0.55)

-1.00 (-1.60, -0.40)

-0.20 (-0.47, 0.07)

-0.50 (-1.02, 0.02)

0.00 (-0.81, 0.81)

0.40 (-0.07, 0.87)

-0.50 (-0.74, -0.26)

-0.11 (-0.57, 0.36)

-0.24 (-0.55, 0.06)

-0.20 (-1.31, 0.91)

2477

13, 39.3 (1.5)

**N, mean**

96

26, 37 (2.6)

764, 38.4 (2.34)

30, 39.3 (.59)

151, 39.1 (2.33)

507, 38.9 (2.5)

71, 38.6 (2.57)

251, 39 (2)

126, 38.8 (3)

83, 38.6 (3.4)

124, 39.9 (1.8)

293, 37.9 (2.1)

1050

1331

38, 37.6 (2.7)

906476

414, 39.1 (2.2)

**N, mean**

534

66, 37.4 (3.9)

**(SD); Control**

764, 38.3 (2.92)

60, 39.1 (1.56)

151, 39.7 (2.13)

301, 38.7 (2.4)

57970, 39.6 (2.14)

1577, 39.2 (2.3)

188500, 39.3 (1.9)

120, 38.6 (1.9)

124, 39.5 (2)

656353, 38.4 (2)

845278

60664

76, 37.8 (3.1)

100.00

4.64

**%**

9.55

2.21

**Weight**

11.49

8.84

8.07

10.29

6.91

11.39

7.81

4.91

8.48

11.83

38.40

52.05

3.14

-0.16 (-0.38, 0.06)

0.20 (-0.64, 1.04)

0.10 (-0.49, 0.68)

-0.40 (-1.77, 0.97)

**WMD (95% CI)**

0.07 (-0.20, 0.34)

0.23 (-0.22, 0.68)

-0.60 (-1.10, -0.10)

0.20 (-0.15, 0.55)

-1.00 (-1.60, -0.40)

-0.20 (-0.47, 0.07)

-0.50 (-1.02, 0.02)

0.00 (-0.81, 0.81)

0.40 (-0.07, 0.87)

-0.50 (-0.74, -0.26)

-0.11 (-0.57, 0.36)

-0.24 (-0.55, 0.06)

-0.20 (-1.31, 0.91)

2477

13, 39.3 (1.5)

96

26, 37 (2.6)

**(SD); Surgery**

764, 38.4 (2.34)

30, 39.3 (.59)

151, 39.1 (2.33)

507, 38.9 (2.5)

71, 38.6 (2.57)

251, 39 (2)

126, 38.8 (3)

83, 38.6 (3.4)

124, 39.9 (1.8)

293, 37.9 (2.1)

1050

1331

38, 37.6 (2.7)

0

-1.5

0

1.5

Decreased after bariatric surgery Increased after bariatric surgery

Association between maternal bariatric surgery and gestational age (weeks). Studies are presented as: Author, year. Results are subgrouped by type of surgery. WMD=weighted mean difference (weeks). N=total group size. SD=standard deviation. RYGB=Roux-en-Y gastric bypass. BPD=biliopancreatic diversion. LAGB=laparoscopic adjustable gastric banding.
