# Supplementary material for: Pregnancy after bariatric surgery and adverse perinatal outcomes: A systematic review and meta-analysis
Source: PLoS Med. 2019 Aug 6;16(8):e1002866. doi: 10.1371/journal.pmed.1002866 (PMC6684044; doi:10.1371/journal.pmed.1002866)
Supplement: S4 Fig — (DOCX) [file pmed.1002866.s010.docx]

# S4 Figure. Small for gestational age after bariatric surgery meta-analysis

# S4A Figure. Small for gestational age meta-analysis with all studies together

NOTE: Weights are from random effects analysis

Overall (I-squared = 47.0%, p = 0.009)

Ducarme et al. 2007

Hammeken et al. 2017

**Small for gestational age**

Rottenstreich et al. 2018

Marceau et al. 2004

Johansson et al. 2015

Parker et al. 2016

Feichtinger et al. 2016

Roos et al. 2013

Parent et al. 2017

Chevrot et al. 2016

Josefsson et al. 2011

Kjaer et al. 2013

Dixon et al. 2005

Weintraub et al. 2008

Adams et al. 2015

Machado et al. 2017

Wax et al. 2008

Lesko and Peaceman, 2012

Dell'Agnolo et al. 2011

Patel et al. 2008

Lapolla et al. 2010

2.13 (1.80, 2.52)

0.70 (0.09, 5.52)

2.86 (1.09, 7.53)

OR (95% CI)

3.80 (1.35, 10.67)

3.29 (1.64, 6.58)

2.24 (1.71, 2.93)

2.68 (2.16, 3.32)

3.13 (1.20, 8.14)

1.79 (1.46, 2.19)

1.52 (1.31, 1.78)

2.21 (1.06, 4.62)

2.73 (1.27, 5.85)

2.55 (1.51, 4.33)

0.69 (0.21, 2.29)

1.72 (0.72, 4.13)

2.30 (1.57, 3.35)

3.35 (0.96, 11.65)

0.84 (0.21, 3.47)

2.21 (0.94, 5.21)

3.15 (0.36, 27.76)

4.17 (0.66, 26.59)

0.28 (0.03, 2.45)

828/9286

1/13

16/151

**Surgery**

**(n/N)**

17/119

15/156

92/590

90/1585

18/64

131/2507

242/1859

24/139

7/126

24/339

5/79

20/507

92/764

7/30

3/38

12/70

8/41

3/26

1/83

9576/401152

44/414

6/151

**Control**

**(n/N)**

5/119

20/638

178/2336

4073/185120

7/63

369/12338

754/8437

12/139

3982/188500

37/1277

7/79

7/301

43/764

5/60

7/76

12/140

1/14

2/66

5/120

100.00

0.64

2.56

**%**

**Weight**

2.29

4.33

11.48

12.87

2.61

13.14

14.32

3.95

3.75

6.26

1.78

3.03

8.97

1.64

1.31

3.12

0.58

0.79

0.58

2.13 (1.80, 2.52)

0.70 (0.09, 5.52)

2.86 (1.09, 7.53)

**OR (95% CI)**

3.80 (1.35, 10.67)

3.29 (1.64, 6.58)

2.24 (1.71, 2.93)

2.68 (2.16, 3.32)

3.13 (1.20, 8.14)

1.79 (1.46, 2.19)

1.52 (1.31, 1.78)

2.21 (1.06, 4.62)

2.73 (1.27, 5.85)

2.55 (1.51, 4.33)

0.69 (0.21, 2.29)

1.72 (0.72, 4.13)

2.30 (1.57, 3.35)

3.35 (0.96, 11.65)

0.84 (0.21, 3.47)

2.21 (0.94, 5.21)

3.15 (0.36, 27.76)

4.17 (0.66, 26.59)

0.28 (0.03, 2.45)

828/9286

1/13

16/151

17/119

15/156

92/590

90/1585

18/64

131/2507

242/1859

24/139

7/126

24/339

5/79

20/507

92/764

7/30

3/38

12/70

8/41

3/26

1/83

1

.2

.5

1

2

5

10

Decreased after bariatric surgery Increased after bariatric surgery

Association between maternal bariatric surgery and small for gestational age. Studies are presented as: Author, year. OR=odds ratio. CI=confidence interval. n=cases of SGA. N=total group size.

**S4B Figure. Small for gestational age meta-analysis with subtotals by control group**

NOTE: Weights are from random effects analysis

.

.

.

.

**ppBMI Matched**

Lesko and Peaceman, 2012 (All)

Kjaer et al. 2013 (All)

Roos et al. 2013 (All)

Adams et al. 2015 (RYGB)

Feichtinger et al. 2016 (RYGB)

Hammeken et al. 2017 (RYGB)

Machado et al. 2017 (RYGB)

Subtotal (I-squared = 0.0%, p = 0.601)

**Obesity**

Dixon et al. 2005 (LAGB)

Ducarme et al. 2007 (LAGB)

Patel et al. 2008 (RYGB)

Lapolla et al. 2010 (LAGB)

Johansson et al. 2015 (All)

Chevrot et al. 2016 (All)

Parker et al. 2016 (RYGB)

Rottenstreich et al. 2018 (SG)

Subtotal (I-squared = 40.9%, p = 0.106)

**General Population**

Wax et al. 2008 (RYGB)

Josefsson et al. 2011 (All)

Parent et al. 2017 (All)

Subtotal (I-squared = 30.2%, p = 0.239)

**Before Surgery**

Marceau et al. 2004 (BPD)

Weintraub et al. 2008 (All)

Dell'Agnolo et al. 2011 (All)

Subtotal (I-squared = 0.0%, p = 0.512)

**Small for gestational age**

2.21 (0.94, 5.21)

2.55 (1.51, 4.33)

1.79 (1.46, 2.19)

2.30 (1.57, 3.35)

3.13 (1.20, 8.14)

2.86 (1.09, 7.53)

3.35 (0.96, 11.65)

2.03 (1.73, 2.38)

0.69 (0.21, 2.29)

0.70 (0.09, 5.52)

4.17 (0.66, 26.59)

0.28 (0.03, 2.45)

2.24 (1.71, 2.93)

2.21 (1.06, 4.62)

2.68 (2.16, 3.32)

3.80 (1.35, 10.67)

2.21 (1.63, 3.00)

0.84 (0.21, 3.47)

2.73 (1.27, 5.85)

1.52 (1.31, 1.78)

1.65 (1.10, 2.47)

3.29 (1.64, 6.58)

1.72 (0.72, 4.13)

3.15 (0.36, 27.76)

2.59 (1.53, 4.39)

OR (95% CI)

12/70

24/339

131/2507

92/764

18/64

16/151

7/30

300/3925

5/79

1/13

3/26

1/83

92/590

24/139

90/1585

17/119

233/2634

3/38

7/126

242/1859

252/2023

15/156

20/507

8/41

43/704

**(n/N)**

**Surgery**

12/140

37/1277

369/12338

43/764

7/63

6/151

5/60

479/14793

7/79

44/414

2/66

5/120

178/2336

12/139

4073/185120

5/119

4326/188393

7/76

3982/188500

754/8437

4743/197013

20/638

7/301

1/14

28/953

**(n/N)**

**Control**

3.50

9.24

61.88

18.14

2.82

2.76

1.66

100.00

5.66

2.07

2.54

1.89

32.51

12.24

35.87

7.24

100.00

7.42

20.77

71.81

100.00

57.67

36.46

5.87

100.00

**Weight**

**%**

2.21 (0.94, 5.21)

2.55 (1.51, 4.33)

1.79 (1.46, 2.19)

2.30 (1.57, 3.35)

3.13 (1.20, 8.14)

2.86 (1.09, 7.53)

3.35 (0.96, 11.65)

2.03 (1.73, 2.38)

0.69 (0.21, 2.29)

0.70 (0.09, 5.52)

4.17 (0.66, 26.59)

0.28 (0.03, 2.45)

2.24 (1.71, 2.93)

2.21 (1.06, 4.62)

2.68 (2.16, 3.32)

3.80 (1.35, 10.67)

2.21 (1.63, 3.00)

0.84 (0.21, 3.47)

2.73 (1.27, 5.85)

1.52 (1.31, 1.78)

1.65 (1.10, 2.47)

3.29 (1.64, 6.58)

1.72 (0.72, 4.13)

3.15 (0.36, 27.76)

2.59 (1.53, 4.39)

**OR (95% CI)**

12/70

24/339

131/2507

92/764

18/64

16/151

7/30

300/3925

5/79

1/13

3/26

1/83

92/590

24/139

90/1585

17/119

233/2634

3/38

7/126

242/1859

252/2023

15/156

20/507

8/41

43/704

Decreased after bariatric surgery

Increased after bariatric surgery

1

.2

.5

1

2

5

10

2.

Association between maternal bariatric surgery and small for gestational age. Studies are presented as: Author, year (type of bariatric surgery). Results are subgrouped by control group. n=cases of SGA. N=total group size. OR=odds ratio. CI=confidence interval. ppBMI=pre-pregnancy body mass index. All=all bariatric surgery. RYGB=Roux-en-Y gastric bypass. LAGB=laparoscopic adjustable gastric banding. SG=sleeve gastrectomy.
