# Supplementary material for: Pregnancy after bariatric surgery and adverse perinatal outcomes: A systematic review and meta-analysis
Source: PLoS Med. 2019 Aug 6;16(8):e1002866. doi: 10.1371/journal.pmed.1002866 (PMC6684044; doi:10.1371/journal.pmed.1002866)
Supplement: S5 Fig — (DOCX) [file pmed.1002866.s011.docx]

# S5 Figure. Large for gestational age after bariatric surgery meta-analysis

# S5A Figure. Large for gestational age meta-analysis with all studies together

NOTE: Weights are from random effects analysis

Overall (I-squared = 69.5%, p = 0.000)

Hammeken et al. 2017

Wax et al. 2008

Ducarme et al. 2007

Johansson et al. 2015

Dixon et al. 2005

Feichtinger et al. 2016

Lapolla et al. 2010

Weintraub et al. 2008

Josefsson et al. 2011

Parker et al. 2016

Burke et al. 2010

Lesko and Peaceman, 2012

**Large for gestational age**

Rottenstreich et al. 2018

Kjaer et al. 2013

Parent et al. 2017

Roos et al. 2013

Wittgrove et al. 1998

Chevrot et al. 2016

Adams et al. 2015

Marceau et al. 2004

Patel et al. 2008

0.42 (0.34, 0.54)

0.14 (0.02, 1.13)

0.39 (0.02, 8.26)

0.49 (0.06, 3.85)

0.33 (0.24, 0.44)

0.60 (0.24, 1.47)

0.18 (0.02, 1.62)

0.61 (0.30, 1.21)

0.39 (0.20, 0.76)

1.01 (0.37, 2.74)

0.60 (0.47, 0.76)

0.72 (0.49, 1.06)

0.30 (0.09, 1.07)

OR (95% CI)

0.07 (0.02, 0.31)

0.31 (0.15, 0.64)

0.73 (0.60, 0.90)

0.56 (0.45, 0.69)

0.13 (0.03, 0.72)

0.44 (0.22, 0.86)

0.30 (0.20, 0.46)

0.16 (0.08, 0.29)

0.13 (0.01, 2.33)

529/9605

1/151

0/38

1/13

51/590

9/79

1/64

15/83

16/507

4/126

70/1585

59/354

3/70

2/119

8/339

122/1859

105/2507

2/36

15/139

33/764

12/156

0/26

**Surgery**

22109/401447

7/151

2/76

60/414

523/2336

14/79

5/63

32/120

23/301

5908/188500

13329/185120

75/346

18/140

**(n/N)**

23/119

93/1277

736/8437

895/12338

7/23

30/139

99/764

222/638

8/66

**Control**

100.00

1.08

0.54

1.13

8.90

4.05

1.02

5.44

5.71

3.57

9.44

8.20

2.56

**Weight**

2.01

5.12

9.75

9.69

1.61

5.58

7.92

6.07

0.61

**%**

0.42 (0.34, 0.54)

0.14 (0.02, 1.13)

0.39 (0.02, 8.26)

0.49 (0.06, 3.85)

0.33 (0.24, 0.44)

0.60 (0.24, 1.47)

0.18 (0.02, 1.62)

0.61 (0.30, 1.21)

0.39 (0.20, 0.76)

1.01 (0.37, 2.74)

0.60 (0.47, 0.76)

0.72 (0.49, 1.06)

0.30 (0.09, 1.07)

**OR (95% CI)**

0.07 (0.02, 0.31)

0.31 (0.15, 0.64)

0.73 (0.60, 0.90)

0.56 (0.45, 0.69)

0.13 (0.03, 0.72)

0.44 (0.22, 0.86)

0.30 (0.20, 0.46)

0.16 (0.08, 0.29)

0.13 (0.01, 2.33)

529/9605

1/151

0/38

1/13

51/590

9/79

1/64

15/83

16/507

4/126

70/1585

59/354

3/70

**(n/N)**

2/119

8/339

122/1859

105/2507

2/36

15/139

33/764

12/156

0/26

1

.05

.1

.2

.5

1

2

3

Decreased after bariatric surgery Increased after bariatric surgery

Association between maternal bariatric surgery and large for gestational age. Studies are presented as: Author, year. OR=odds ratio. CI=confidence interval. n=cases of LGA. N=total group size.

**S5B Figure: Large for gestational age meta-analysis with subtotals by control group**

NOTE: Weights are from random effects analysis

.

.

.

.

**ppBMI Matched**

Lesko and Peaceman, 2012 (All)

Kjaer et al. 2013 (All)

Roos et al. 2013 (All)

Adams et al. 2015 (RYGB)

Feichtinger et al. 2016 (RYGB)

Hammeken et al. 2017 (RYGB)

Subtotal (I-squared = 54.1%, p = 0.053)

**Obesity**

Dixon et al. 2005 (LAGB)

Ducarme et al. 2007 (LAGB)

Patel et al. 2008 (RYGB)

Burke et al. 2010 (All)

Lapolla et al. 2010 (LAGB)

Johansson et al. 2015 (All)

Chevrot et al. 2016 (All)

Parker et al. 2016 (RYGB)

Rottenstreich et al. 2018 (LAGB/SG)

Subtotal (I-squared = 62.7%, p = 0.006)

**General Population**

Wax et al. 2008 (RYGB)

Josefsson et al. 2011 (All)

Parent et al. 2017 (All)

Subtotal (I-squared = 0.0%, p = 0.756)

**Before Surgery**

Wittgrove et al. 1998 (RYGB)

Marceau et al. 2004 (BPD)

Weintraub et al. 2008 (All)

Subtotal (I-squared = 57.3%, p = 0.096)

**Large for gestational age**

0.30 (0.09, 1.07)

0.31 (0.15, 0.64)

0.56 (0.45, 0.69)

0.30 (0.20, 0.46)

0.18 (0.02, 1.62)

0.14 (0.02, 1.13)

0.37 (0.24, 0.55)

0.60 (0.24, 1.47)

0.49 (0.06, 3.85)

0.13 (0.01, 2.33)

0.72 (0.49, 1.06)

0.61 (0.30, 1.21)

0.33 (0.24, 0.44)

0.44 (0.22, 0.86)

0.60 (0.47, 0.76)

0.07 (0.02, 0.31)

0.47 (0.34, 0.66)

0.39 (0.02, 8.26)

1.01 (0.37, 2.74)

0.73 (0.60, 0.90)

0.74 (0.61, 0.90)

0.13 (0.03, 0.72)

0.16 (0.08, 0.29)

0.39 (0.20, 0.76)

0.22 (0.11, 0.47)

OR (95% CI)

3/70

8/339

105/2507

33/764

1/64

1/151

151/3895

9/79

1/13

0/26

59/354

15/83

51/590

15/139

70/1585

2/119

222/2988

0/38

4/126

122/1859

126/2023

2/36

12/156

16/507

30/699

**(n/N)**

**Surgery**

18/140

93/1277

895/12338

99/764

5/63

7/151

1117/14733

14/79

60/414

8/66

75/346

32/120

523/2336

30/139

13329/185120

23/119

14094/188739

2/76

5908/188500

736/8437

6646/197013

7/23

222/638

23/301

252/962

**(n/N)**

**Control**

8.56

17.96

37.26

29.37

3.32

3.52

100.00

8.59

2.32

1.23

18.34

11.74

20.09

12.08

21.45

4.16

100.00

0.40

3.80

95.79

100.00

14.86

43.48

41.66

100.00

**Weight**

**%**

0.30 (0.09, 1.07)

0.31 (0.15, 0.64)

0.56 (0.45, 0.69)

0.30 (0.20, 0.46)

0.18 (0.02, 1.62)

0.14 (0.02, 1.13)

0.37 (0.24, 0.55)

0.60 (0.24, 1.47)

0.49 (0.06, 3.85)

0.13 (0.01, 2.33)

0.72 (0.49, 1.06)

0.61 (0.30, 1.21)

0.33 (0.24, 0.44)

0.44 (0.22, 0.86)

0.60 (0.47, 0.76)

0.07 (0.02, 0.31)

0.47 (0.34, 0.66)

0.39 (0.02, 8.26)

1.01 (0.37, 2.74)

0.73 (0.60, 0.90)

0.74 (0.61, 0.90)

0.13 (0.03, 0.72)

0.16 (0.08, 0.29)

0.39 (0.20, 0.76)

0.22 (0.11, 0.47)

**OR (95% CI)**

3/70

8/339

105/2507

33/764

1/64

1/151

151/3895

9/79

1/13

0/26

59/354

15/83

51/590

15/139

70/1585

2/119

222/2988

0/38

4/126

122/1859

126/2023

2/36

12/156

16/507

30/699

Decreased after bariatric surgery

Increased after bariatric surgery

1

.05

.1

.2

.5

1

2

3

Association between maternal bariatric surgery and large for gestational age. Studies are presented as: Author, year (type of bariatric surgery). Results are subgrouped by control group. n=cases of LGA. N=total group size. OR=odds ratio. CI=confidence interval. ppBMI=pre-pregnancy body mass index. All=all bariatric surgery. RYGB=Roux-en-Y gastric bypass. LAGB=laparoscopic adjustable gastric banding. SG=sleeve gastrectomy.
