# Supplementary material for: Pregnancy after bariatric surgery and adverse perinatal outcomes: A systematic review and meta-analysis
Source: PLoS Med. 2019 Aug 6;16(8):e1002866. doi: 10.1371/journal.pmed.1002866 (PMC6684044; doi:10.1371/journal.pmed.1002866)
Supplement: S6 Fig — (DOCX) [file pmed.1002866.s012.docx]

# S6 Figure. Birth weight (grams) after bariatric surgery meta-analysis with subtotals by type of surgery

NOTE: Weights are from random effects analysis

.

.

.

.

Overall (I-squared = 75.7%, p = 0.000)

Goldman et al. 2016

Subtotal (I-squared = 31.1%, p = 0.234)

**LAGB**

Machado et al. 2017

Belogolovkin et al. 2012

Marceau et al. 2004

Josefsson et al. 2011

Stentebjerg et al. 2017

Weintraub et al. 2008

Adams et al. 2015

**All bariatric surgery**

**Birth weight (grams)**

Lapolla et al. 2010

Wax et al. 2008

**BPD**

Hammeken et al. 2017

**RYGB**

Patel et al. 2008

Dixon et al. 2005

Chevrot et al. 2016

Subtotal (I-squared = 0.0%, p = 0.470)

Subtotal (I-squared = 21.2%, p = 0.279)

Gascoin et al. 2017

Skull et al. 2004

Berglind et al. 2014

-242.42 (-307.43, -177.40)

-599.73 (-996.16, -203.30)

-135.14 (-289.17, 18.90)

-206.31 (-370.12, -42.50)

-282.00 (-344.53, -219.47)

-500.00 (-570.85, -429.15)

-163.50 (-288.46, -38.54)

-220.00 (-354.77, -85.23)

-185.00 (-268.76, -101.24)

-200.00 (-263.70, -136.30)

WMD (95% CI)

-195.00 (-375.12, -14.88)

-248.00 (-525.15, 29.15)

-266.98 (-404.09, -129.87)

-93.00 (-483.19, 297.19)

47.00 (-204.14, 298.14)

-211.00 (-332.55, -89.45)

-226.10 (-273.43, -178.78)

-223.71 (-273.68, -173.74)

-350.00 (-537.01, -162.99)

-220.00 (-463.32, 23.32)

-200.00 (-362.27, -37.73)

2799

12, 2984 (510)

211

30, 3129 (271)

293, 2991 (546)

251, 3000 (500)

126, 3355 (716)

71, 3225 (579)

507, 3079 (567)

764, 3092 (568)

**(SD); Surgery**

83, 3178 (705)

38, 2896 (636)

151, 3232 (620)

26, 2951 (646)

79, 3397 (545)

139, 3317 (520)

1148

1189

56, 3000 (570)

49, 3310 (600)

124, 3500 (600)

**N, mean**

906354

14, 3584 (519)

230

60, 3335 (521)

656353, 3273 (556)

1577, 3500 (700)

188500, 3519 (570)

57970, 3445 (611)

301, 3264 (599)

764, 3292 (696)

**(SD); Control**

120, 3373 (543)

76, 3144 (843)

151, 3499 (595)

39, 3044 (959)

79, 3350 (1000)

139, 3528 (514)

59130

845417

56, 3350 (430)

31, 3530 (500)

124, 3700 (700)

**N, mean**

100.00

2.09

13.48

5.91

8.71

8.51

7.02

6.73

8.18

8.68

**Weight**

5.48

3.48

6.66

2.15

3.92

7.12

41.02

36.98

5.31

4.07

5.96

**%**

-242.42 (-307.43, -177.40)

-599.73 (-996.16, -203.30)

-135.14 (-289.17, 18.90)

-206.31 (-370.12, -42.50)

-282.00 (-344.53, -219.47)

-500.00 (-570.85, -429.15)

-163.50 (-288.46, -38.54)

-220.00 (-354.77, -85.23)

-185.00 (-268.76, -101.24)

-200.00 (-263.70, -136.30)

**WMD (95% CI)**

-195.00 (-375.12, -14.88)

-248.00 (-525.15, 29.15)

-266.98 (-404.09, -129.87)

-93.00 (-483.19, 297.19)

47.00 (-204.14, 298.14)

-211.00 (-332.55, -89.45)

-226.10 (-273.43, -178.78)

-223.71 (-273.68, -173.74)

-350.00 (-537.01, -162.99)

-220.00 (-463.32, 23.32)

-200.00 (-362.27, -37.73)

2799

12, 2984 (510)

211

30, 3129 (271)

293, 2991 (546)

251, 3000 (500)

126, 3355 (716)

71, 3225 (579)

507, 3079 (567)

764, 3092 (568)

83, 3178 (705)

38, 2896 (636)

151, 3232 (620)

26, 2951 (646)

79, 3397 (545)

139, 3317 (520)

1148

1189

56, 3000 (570)

49, 3310 (600)

124, 3500 (600)

0

-700

-500

-200

0

200

Decreased after bariatric surgery Increased after bariatric surgery

Association between maternal bariatric surgery and birth weight (grams). Studies are presented as: Author, year. Results are subgrouped by type of surgery. WMD=weighted mean difference (grams). N=total group size. SD=standard deviation. RYGB=Roux-en-Y gastric bypass. BPD=biliopancreatic diversion. LAGB=laparoscopic adjustable gastric banding.
