# Supplementary material for: Pregnancy after bariatric surgery and adverse perinatal outcomes: A systematic review and meta-analysis
Source: PLoS Med. 2019 Aug 6;16(8):e1002866. doi: 10.1371/journal.pmed.1002866 (PMC6684044; doi:10.1371/journal.pmed.1002866)
Supplement: S7 Fig — (DOCX) [file pmed.1002866.s013.docx]

# S7 Figure. Funnel plots of publication bias for perinatal outcomes after bariatric surgery

| A) Perinatal mortality   | B) Congenital anomalies   |
| --- | --- |
| C) Pre-term birth   | D) Post-term birth   |
| E) Small for gestational age   | F) Large for gestational age   |
| G) NICU admission   | H) Birth weight (grams)   |
| I) Gestational age (weeks)   | |

Funnel plots of study size against effect size. Asymmetrical funnel plots indicate publication bias. The red line represents the line no effect and the black line represents the effect size calculated from meta-analyses.
