# Supplementary material for: Pregnancy after bariatric surgery and adverse perinatal outcomes: A systematic review and meta-analysis
Source: PLoS Med. 2019 Aug 6;16(8):e1002866. doi: 10.1371/journal.pmed.1002866 (PMC6684044; doi:10.1371/journal.pmed.1002866)
Supplement: S8 Fig — (DOCX) [file pmed.1002866.s014.docx]

# S8 Figure. Crude vs. adjusted data for studies reporting adjusted odds ratios

## S8A Figure. Pre-term birth meta-analysis: crude vs. adjusted pooled odds ratio


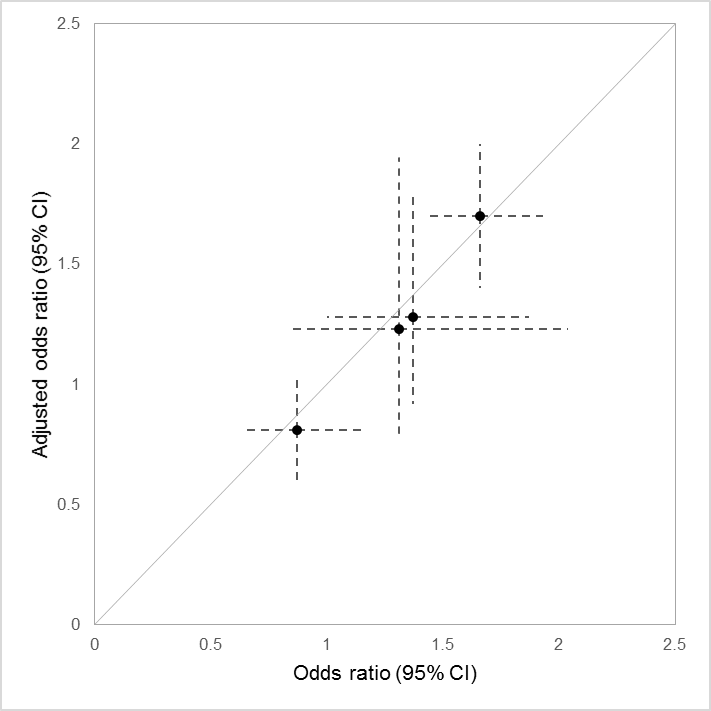


Crude odds ratio vs. adjusted odds ratios (with 95% confidence intervals) for the four studies which provided adjusted odds ratios for bariatric surgery and pre-term birth [Kjaer *et al.* 2013, Roos *et al.* 2013, Adams *et al.* 2015, Johansson *et al.* 2015]. The solid grey line represents the line of equality.

## S8B Figure. Post-term birth meta-analysis: crude vs. adjusted pooled odds ratio


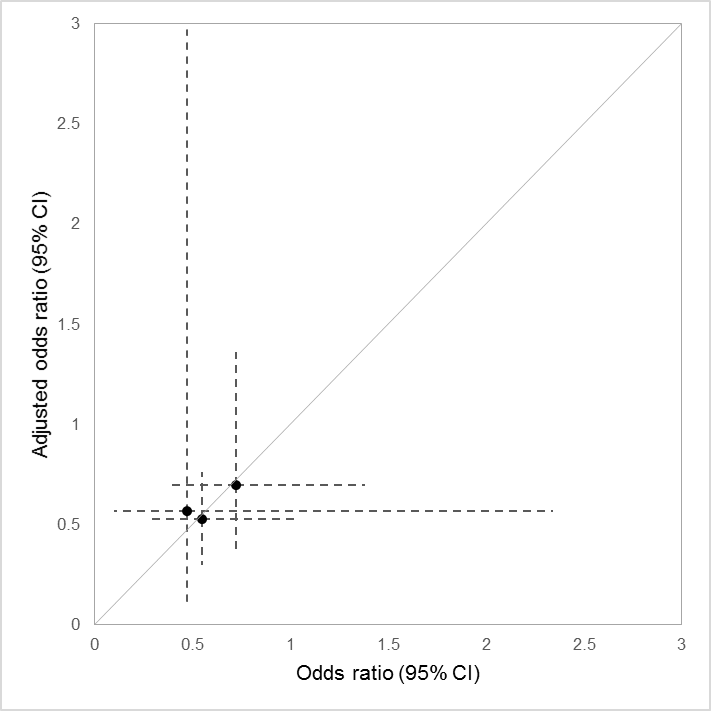


Crude odds ratio vs. adjusted odds ratios (with 95% confidence intervals) for the three studies which provided adjusted odds ratios for bariatric surgery and post-term birth [Wax *et al.* 2008, Kjaer *et al.* 2013, Adams *et al.* 2015]. The solid grey line represents the line of equality.

## S8C Figure. Large for gestational age meta-analysis: crude vs. adjusted pooled odds ratio


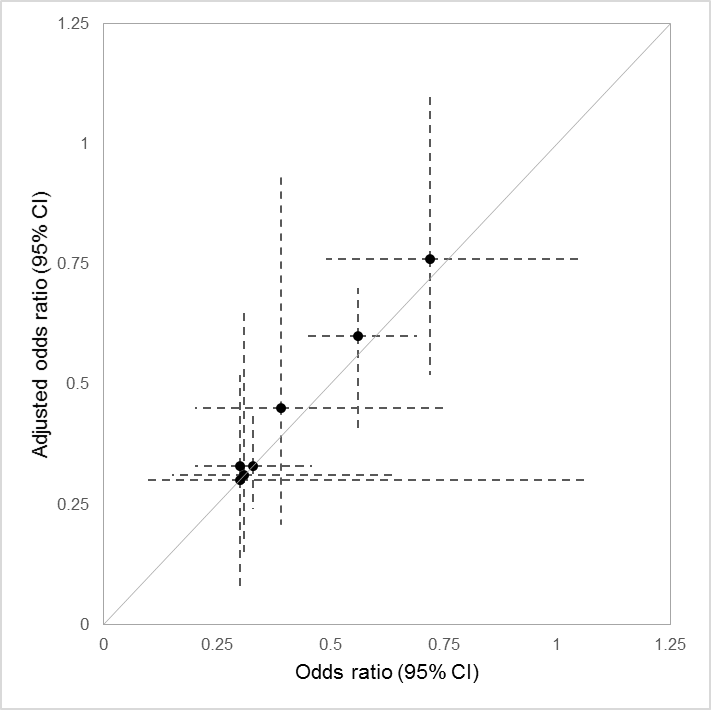


Crude odds ratio vs. adjusted odds ratios (with 95% confidence intervals) for the seven studies which provided adjusted odds ratios for bariatric surgery and large for gestational age [Weintraub *et al.* 2008, Burke *et al.* 2010, Lesko and Peaceman 2012, Kjaer *et al.* 2013, Roos *et al.* 2013, Adams *et al.* 2015, Johansson *et al.* 2015]. The solid grey line represents the line of equality.

## S8D Figure. Small for gestational age meta-analysis: crude vs. adjusted pooled odds ratio


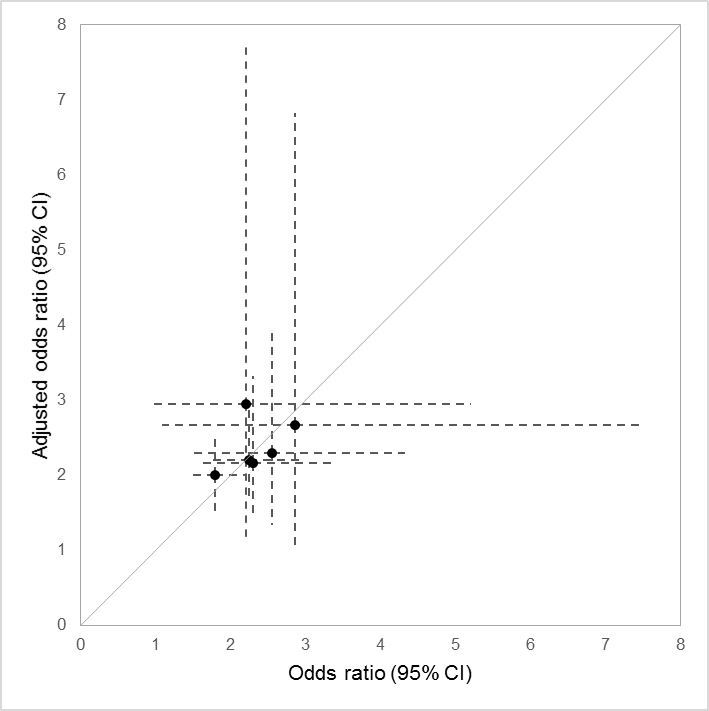


Crude odds ratio vs. adjusted odds ratios (with 95% confidence intervals) for the six studies which provided adjusted odds ratios for bariatric surgery and small for gestational age [Lesko and Peaceman 2012, Kjaer *et al.* 2013, Roos *et al.* 2013, Adams *et al.* 2015, Johansson *et al.* 2015, Hammeken *et al.* 2017]. The solid grey line represents the line of equality.

## S8E Figure. Congenital anomalies meta-analysis: crude vs. adjusted pooled odds ratio


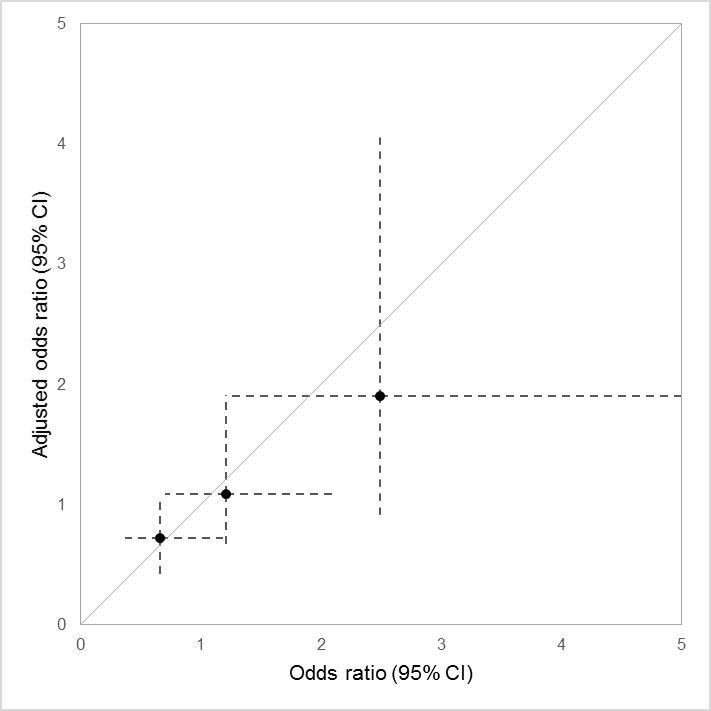


Crude odds ratio vs. adjusted odds ratios (with 95% confidence intervals) for the three studies which provided adjusted odds ratios for bariatric surgery and congenital anomalies [Weintraub *et al.* 2008, Josefsson *et al.* 2013, Johansson *et al.* 2015]. The solid grey line represents the line of equality.
